# Bio-Monitoring of Metal(loid)s Pollution in Dry Riverbeds Affected by Mining Activity

**DOI:** 10.3390/plants12213775

**Published:** 2023-11-05

**Authors:** José Cuevas, Ángel Faz, Silvia Martínez-Martínez, María Gabarrón, Juan Beltrá, Jacinto Martínez, José A. Acosta

**Affiliations:** Sustainable Use, Management and Reclamation of Soil and Water Research Group, Universidad Politécnica de Cartagena, Paseo Alfonso XIII 48, 30203 Cartagena, Spain; jose.cuevas@upct.es (J.C.); angel.fazcano@upct.es (Á.F.); silvia.martinez@upct.es (S.M.-M.); m.gabarron@upct.es (M.G.); juan.beltra@upct.es (J.B.); jacinto.martinez@upct.es (J.M.)

**Keywords:** Mar Menor lagoon, bio-monitoring, native plant, dry riverbed, phytotoxic

## Abstract

The aim of this study was to evaluate the most abundant native plants that could be used as a bio-monitor of metal(loid) concentration in dry riverbeds affected by mining activities. Three plants species and their respective rhizospheric soils were sampled from the El Beal (*Piptatherum miliaceum*, 15 samples), La Carrasquilla (*Foeniculum vulgare*, 10 samples), and Ponce (*Dittrichia viscosa*, 12 samples) dry riverbeds from the mining district of Cartegena-La Unión (SE Spain). There is scanty bibliography of the capacity of these species to be used as bio-monitors in the dry riverbeds. Plants categorized as a bio-monitor were established according to the bioaccumulation factor (BF), mobility ratio (MR), and linear correlations between metal(loid) concentrations in plants tissues (root or stem)-rhizospheric soils. The rhizospheric soils were highly contaminated for As, Cd, Pb, and Zn (Cf ≥ 6), and moderately contaminated for Mn (1 ≤ Cf < 3). *Piptatherum miliaceum* presented on Cd similar mean concentrations on rhizospheric soil and root, BF = 1.07, with a strong correlation soil–root (r = 0.61, *p* = 0.02). Therefore, of the three species with the capacity to grow in the area, *Piptatherum miliaceum* showed characteristics to be considered as a bio-monitor for Cd, with a BF > 1, and a positive–significant correlation between the rhizospheric soil and roots.

## 1. Introduction

Bio-indicators and bio-monitors are flora and fauna members or groups of them which are used indirectly to measure or identify metal contaminants in their environment, being systematically collected and analyzed for potential health hazards to biota and humans [1]. A bio-indicator is an organism or a part of one that contains information on the quality of the environment. However, a bio-monitor is an organism or a part of one that contains information on the quantitative aspects of the quality of the environment. In Europe and North America, vascular plants, mosses, and lichens have been used as bio-monitors in order to estimate the environmental pollution by toxic metals [2]. The use of plants tissues (leaves, roots, and stems) as bio-indicators and bio-monitors has shown efficiency for environmental characterization due to their capacity to retain or absorb heavy metal pollution from the soil [3]. The accumulation of heavy metals in plant leaves is influenced by multiple factors, including proximity to the source of pollution and variations in plant anatomy and physiology.

Plants that are adapted to grow under dry conditions, with low soil nutrient content and high heavy metal concentrations, can be ideal to be used as in situ bio-indicators or bio-monitors for metal contamination [4]. Plants used as bio-monitors of heavy metals are measured during the vegetative period of the year, in which the specimens are collected. However, heavy metal concentrations accumulated on a yearly basis are also extremely important [5]. Bio-indicator plant species are efficient, highly specific, and low cost with respect to conventional methodologies [6]. Many plants have been accepted as indicators for bio-monitoring of the heavy metal pollution in the environment [7,8,9]. However, the use of these species is conditioned to their adaptation to the climate where the contamination occurs. As a matter of fact, the presence or absence of certain plants can offer significant insights into the contamination of the environment [10]. As a result, it is necessary to carry out specific studies in each place to identify the potential plant for use. 

Improper management of abandoned mines can cause leaching of heavy metals into the soil and groundwater [11]. Especially, mine tailings that contain high concentrations of mobile contaminants represent an ecological risk for flora and fauna in the surrounding areas. Bioaccumulation of different metals in the ecosystem is of great concern as it can be a potential risk for damaging plant growth and productivity [12]. Nevertheless, some metals are essential elements for a wide range of organisms and play structural roles. 

Soils from mining areas often have geochemical properties that inhibit plant establishment, including low pH, high salt content, and low organic matter, aside from high toxic metal(loid) concentrations. Additionally, soils from these areas have inappropriate physical conditions (soil compaction, water retention, and drainage) [13]. The spontaneous vegetation in heavily polluted mining areas results from strong tolerance mechanisms that allow these plants to grow under the stressful conditions prevailing at these sites [14]. However, there is a lack of studies using spontaneous vegetation (native plants) as a potential spatial bio-monitor in soil contaminated by metal(loid)s.

Metal-tolerant plants that grow on or around mining areas in semi-arid regions have been previously studied [15]. Especially those capable of spontaneously colonizing mining sites and that are well adapted. Therefore, the aim of this study was to evaluate the metal(loid)s’ concentration in rhizospheric soils and plant tissues (root–stem) of spontaneous plant species growing on dry riverbeds affected by mining activities, which allow us to identify those species that may be used as bio-monitors. This study was developed on the dry riverbeds connecting Mar Menor lagoon and the mining district of Cartagena-La Unión (SE, Spain), a zone characterized by abandoned mines and tailing ponds from the past mining operations, being nowadays part of the landscape of the area. Field inspections showed 12 different plants on the studied dry riverbeds, with the most represented being *Piptatherum miliaceum*, *Foeniculum vulgare*, and *Dittrichia viscosa*. Several studies in the Mediterranean have shown that *Piptatherum miliaceum*, with a high heavy-metal accumulation in its root system, could be considered as a phytostabilizer plant species for Pb [15,16], therefore, it may be used to revegetate soils with high heavy metal concentrations in semi-arid climates. There is scanty bibliography on the metal(loid) accumulation capacity of *Foeniculum vulgare*. However, this species is known for metal adsorption in the stem–leaves and has shown a higher amount in the stem than in the leaves, especially for Pb. Additionally, *Foeniculum vulgare* have exhibited a great potential as a hyperaccumulator of As, Cu, Ni, Sb, Sn, and Zn [17]. *Dittrichia viscosa* have been reported to be colonizers of mining wastes in the Mediterranean area by Pérez-Sirvent et al. [18]. Few studies have described *Dittrichia viscosa* as a good candidate for phytoremediation purposes. In Italy, this species was found to be growing spontaneously in a former Zn-smelter plant [19] and on an abandoned mining site [20].

Although these three selected plant species, *Piptatherum miliaceum*, *Foeniculum vulgare*, and *Dittrichia viscosa*, have been widely studied for phytomanagement purposes, they have not been studied as a potential biomonitor species, establishing a correlation among the metal(loid) concentration in the soil and the concentration of these metal(loid)s in plant tissues, especially in the polluted dry riverbeds. These data will provide essential information for monitoring, controlling, and managing of metal(loid) pollution in dry riverbeds, to prevent risks from ecological damage. 

## 2. Materials and Methods 

### 2.1. Study Area 

**Figure 1:**
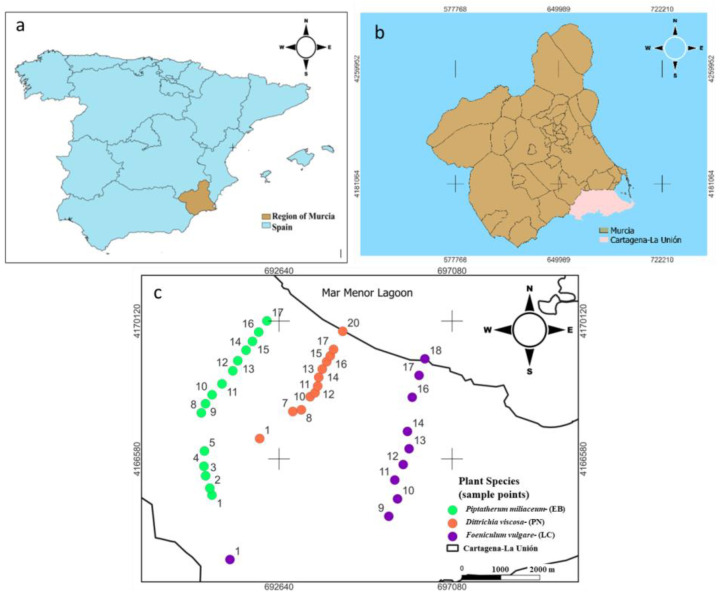
Study area and sampling points for the most abundant plant species selected on each dry riverbed. (**a**) Map fof the region of Murcia (Spain); (**b**) map of the study area Cartagena La Unión mining district; (**c**) sampling points for the most abundant plant species selected on each dry riverbed.

The study was conducted in the Sierra Minera Cartagena-La Unión (Region of Murcia, SE Spain) (Figure 1), which is one of the oldest mining districts in Europe, where metal mining started 2500 years ago until its closure in 1991 [21]. The main minerals extracted were pyrite (Fe), galena (Pb), and sphalerite (Zn) [22]. As a consequence of this activity, more than 85 mining ponds are rich in toxic elements such as As, Pb, and Cd. 

In the Cartagena-La Unión Mining District there are several unconnected dry riverbeds, three of them are El Beal (EB), La Carrasquilla (LC), and Ponce (PN) (Figure 1), which are responsible for Mar Menor coastal lagoon contamination from the mining area (Figure 1), transporting water and mining sediments after heavy rainfall episodes. The climate of the area is semi-arid Mediterranean with a mean annual temperature of 18 °C, annual rainfall of 275 mm, and a potential evapotranspiration rate surpasses 900 mm year ^−1^ [23]. The soils of this area present poor physical structure, low organic matter content, and scarce plant density, which favor wind–water erosion of waste materials currently stored in abandoned tailing ponds. In addition, the semi-arid climate of this area makes the establishment and development of a plant cover relatively difficult [14]. Few studies have been carried out on the effect of mining activity on the dry riverbeds of this mining district, Cuevas et al. [24] evaluated the level of pollution, sources, and potential risk of heavy metals (Zn, Cu, Mn, Cd, Cr, Ni, Fe, and Pb) and arsenic (As) in four dry riverbeds affected by mine tailing from Cartagena- La Union mining district using principal component analysis (PCA), hierarchical cluster analysis (HCA), the contamination factor (Cf), the pollution load index (PLI), and the potential ecological risk index (RI). In addition, Martinez-Lopez et al. [25] assessed the solubility and bioavailability of arsenic in the soils of the different dry riverbeds in the same area, in order to establish possible relationships between arsenic content and the soil mineralogical composition. Some plant species growing in this area show relative tolerance to high heavy metal concentrations [26]. The natural vegetation is mainly based on thicket plant species with xerophytic characteristics [21]. A recent study performed by Parraga-Aguado et al. [27] showed in this mining area that the soil pH and salinity were the main factors driving the plant distribution in mine tailings. These authors suggested that high evaporation rates, typical of this semi-arid area, might have favored the existence of high salinity and low fertility. However, there are not any studies of the vegetation growing on dry riverbeds, which connect the mining area with Mar Menor Lagoon. 

### 2.2. Sampling Collection 

The location of the samplings covered the entire surface of each dry riverbed from its headwaters to its mouth. The sample point area along the surface was previously stablished by GPS coordinates. During the field inspection, an area of 10 × 10 m^2^ around the coordinate location with the most abundant visualized species was sampled on each point. The El Beal (EB) dry riverbed has a total area of 7.60 km^2^ and has a fluvial length of 7.20 km. The vertical elevation variation for this riverbed stands at 242 m, and it exhibits an average gradient of approximately 1.90%. The La Carrasquilla (LC) dry riverbed covers a more extensive area of 29.0 km^2^ and has a length 10.6 km. This riverbed reveals an elevation disparity of 232 m and has a prevailing slope of 1.30%. The Ponce (PN) dry riverbed encompasses an area of 11.9 km^2^ and has a length of 6.40 km. The altitude variation for the PN riverbed is notably higher at 384 m, and its predominant gradient is 3.10% [24].

For the three species selected, *Piptatherum miliaceum* (EB), *Foeniculum vulgare* (LC), and *Dittrichia viscosa* (PN), the total samples collected were EB (15 samples), LC (10 samples), and PN (12 samples) (Figure 1). Each sample of the selected plants was composed of 3 replicates, where the plants were taken from the root, including rhizospheric soil, which was carefully separated from the roots with a spade and placed in airtight bags for transportation to the laboratory. *Piptatherum miliaceum* (cosson or smilo grass) species grows naturally in polluted ecosystems [15]. It is commonly found in pastures and open grassy places throughout the Mediterranean region and has been introduced worldwide [16]. This plant has a strong root system which facilitates a suitable fixation of the plant in the soil and reduces erosion [28]. Rooted species can absorb metals through their roots, rhizomes, and leaves. The last one provides an expanded area to trap particulate matter, sorb metal ions, and accumulate and sequester pollutants [29].

*Foeniculum vulgare* (fennel) is native to Southern Europe and the Mediterranean region, preferring loamy soil, rich in organic matter with a pH between 6.5 and 8.0 and soil temperature between 10 and 24 °C [30]. It is a highly aromatic [31] and flavorful herb with culinary, cosmetic [32], and medicinal uses [33]. According to other studies, it could be effective in phytoremediation [34]. It belongs to *Foeniculum vulgare* and can be cultivated or grown easily in contaminated soils and waste substrates. 

*Dittrichia viscosa* (greuter, stinkwort, or false yellowhead) is a common perennial herbaceous plant in Mediterranean regions; it is abundant in anthropically altered areas [35]. It grows rapidly and is well adapted to a wide range of environmental stresses [36]. *Dittrichia viscosa* is characterized by a quite substantial root system and the ratio between below- and above-ground biomass is 0.24 [37]. It can colonize highly contaminated soils, showing significant potential in soil management. Moreover, it can be used for phytoremediation in mining-affected semi-arid soils, since it is an efficient bioaccumulator of trace metals [38]. This species showed a high ratio of heavy metal concentrations in shoots to that in roots [39]. 

### 2.3. Rhizospheric Soil Analyses 

Rhizospheric soil samples were dried in an oven until constant in weight, then sieved through 2 mm grain size. The pH was determined by a 1:2.5 rhizospheric soil:water ratio and a 1:5 ratio for electrical conductivity (EC) [40]. Soil particle size was determined by MASTERSIZER 2000LF (Thermo Fisher Scientific, Waltham, MA, USA) and classified by the USDA triangle method [40]. The concentrations of total carbon (CT) and total nitrogen (NT) were quantified using an analyzer LECO CHN 628. Equivalent calcium carbonate (CI) was determined using a Bernard calcimeter [41]. Organic carbon (OC) concentration was calculated from the difference between total carbon (TC) and inorganic carbon (IC). Water soluble calcium (Ca^2+^), magnesium (Mg^2+^), sodium (Na^+^), chloride (Cl^−^), nitrate (NO_3_^−^), and sulfate (SO_4_^2−^) were measured by 861 METROHM ion chromatography system, using deionized water in a 1:5 rhizospheric soil:water ratio.

Total, bioavailable, and soluble concentrations of arsenic (As), cadmium (Cd), chromium (Cr), copper (Cu), iron (Fe), manganese (Mn), nickel (Ni), lead (Pb), and zinc (Zn) were determined by inductively coupled plasma spectrometer (ICP-MS Perkinelmer optima 8300-DV) [42]. For total concentration, 0.5 g of ground soil was weighed, then digested in Teflon vessels with nitric acid (HNO_3_^−^) and hydrochloric acid (HCl). After completion of digestion, the samples were cooled and filtered (US-EPA 3051). Bioavailable metals for pH > 6, the extractant was diethylenetriaminepentaacetic acid (DTPA) in a 1:2 ratio and, if pH < 6, ethylenediaminetetraacetic acid (EDTA) in a 1:5 ratio [43]. The water-soluble metals were obtained using a 1:5 ratio rhizospheric soil:Milli-Q water. Certified reference material (BAM-U110) from the Federal Institute for Materials Research and Testing [44] and reagent blanks were used as the quality control samples during the analysis. The recovery of metals in the analysis was within <5.0% compared to this reference sample. 

### 2.4. Plant Analyses 

The roots and stems of sampled plants were separated and carefully washed with distilled water to remove all adhering soil particles. The samples were dried in an oven at 50 °C for 72 h and then pulverized with a Retsch Ultra Centrifugal Mill ZM 200. For each sample, 0.5 g was digested in Teflon vessels with nitric acid (HNO_3_) (69%), hydrochloric acid (HCl) (37%), and hydrogen peroxide (H_2_O_2_) (30%). After the completion of digestion, the samples were filtered. Arsenic (As), cadmium (Cd), chromium (Cr), copper (Cu), iron (Fe), manganese (Mn), nickel (Ni), lead (Pb), and zinc (Zn) concentrations were determined by an inductively coupled plasma spectrometer (ICP-MS Perkinelmer optima 8300-DV). 

### 2.5. Metal(loid) Concentration Indices

#### 2.5.1. Contamination Factor

The contamination factor (Cf) indicates the degree of contamination by metal(loid) accumulation in the soil (rhizospheric) on the studied sites with respect to the reference soil [45], following the equation (Formula (1)):

Cf = C_Sample_/C_Background_ (1)

where C_sample_ is the metal concentration in the studied soil (mg kg^−1^) and C_background_ is the concentration in the reference soil (mg kg^−1^). Therefore, this index reflects the anthropogenic input of metal(loid)s in the soil, which is rated from 1 to 6 (low Cf < 1, moderate 1 ≤ Cf < 3, considerable 3 ≤ Cf < 6, and very high Cf ≥ 6) [46]. Local background metal(loid) concentrations for the studied area are (mg kg^−1^): As (7), Cd (0.32), Cr (40.4), Cu (12.6), Ni (21.7), Pb (9.30), and Zn (41.4) [47], and Mn (770) [48]. 

#### 2.5.2. Pollution Load Index

Pollution Load Index (PLI) is obtained as contamination factors (Cfs) for a set of “n” polluting elements [49], and follows the equation (Formula (2)):

PLI = ^n^√(Cf_1_ × Cf_2_ × Cf_3_ ×…Cf_n_) (2)

where Cf_n_ is the contamination factor of each metal, and “n” is the number of metals used in the calculation. A PLI value higher than 1 suggests pollution existence, while values lower than 1 indicate no pollution load. 

#### 2.5.3. Potential Ecological Risk Index

The potential ecological risk index (RI) was used to assess the degree of metal pollution in surface sediment or soil that corresponded to the total concentration, according to the equation proposed by Hakanson [45] (Formulas (3) and (4)).

RI = ∑_i_E_ri_ (3)

E_ri_ = T_ri_ × Cf_i_ (4)

This index is calculated as the sum of the potential risk of each metal (E_ri_), which is the result of multiplying the biological toxic factor of each element (Tri) by its contamination factor (Cf_i_). The T_ri_ was used for Cd, Cr, Cu, Mn, Ni, Pb, Zn, and As: 30, 2, 5, 1, 5, 5, 1, and 10 respectively [50]. The following ranges of RI values were considered in the present study: low ecological risk RI < 150, moderate ecological risk 150 ≤ RI < 300, high ecological risk 300 ≤ RI < 600, and significantly high ecological risk RI ≥ 600 [45]. 

#### 2.5.4. Bioaccumulation Factor (BF)

The bioaccumulation factor (BF) is an index of the ability of the plant to accumulate a metal in the roots with respect to its concentration in the soil [51]. Plant bioaccumulation factor (BF) was calculated by using the equation adopted from Rashed [52] (Formula (5)). The index was used to evaluate the bioavailability (BF_b_). Where C is the concentration of metals (mg kg^−1^) in plant roots and soil (total bioavailability).

BF = C_root_/C_soil_ (5)

#### 2.5.5. Translocation Factor (TF)

The translocation factor (TF) was calculated to determine the relative translocation of metals from the root to the stem of the plant species [53] (Formula (6)).

TF = C_stems_/C_roots_ (6)

where C is the concentration of metals (mg kg^−1^) in the stem and root samples of the selected plant species.

#### 2.5.6. Mobility Ratio (MR)

The mobility ratio (MR) was calculated to evaluate the ability of the plant to accumulate metals in its stems with respect to the metal’s concentration in the soil [54] (Formula (7)). In addition, the index was used to evaluate the bioavailable concentration (MR_b_). Mobility ratio (MR) >1 indicates that the plant is enriched with metals (accumulator), MR = 1 indicates a rather indifferent behavior of the plant towards metals (indicator), and MR < 1 shows that the plant excludes metals from uptake (excluder). Where C is the concentration of metals (mg kg^−1^) in the plant roots and soil (total-bioavailable).

MR = C_stems_/C_soil_ (7)

#### 2.5.7. Statistical and Geostatistical Analysis

Before statistical analysis, data were checked for their normality and homogeneity distribution, and logarithmic transformations were performed when necessary. Correlations between the concentration of metal(loid)s in plant tissues (root–stem) and rhizospheric soils were tested through the Spearman correlation coefficient, considering nine metal(loid)s: As, Cd, Cr, Cu, Fe, Mn, Ni, Pb, and Zn. A correlation coefficient between 0 and 0.19 is considered very weak, 0.2 and 0.39 as weak, 0.40 and 0.59 as moderate, 0.6 and 0.79 as strong, and 0.8 and 1 as a very strong correlation (*p* < 0.05) [55]. All statistics were performed using software SPSS version 23.0 of Statistical Software Package (SPSS Inc., Chicago, IL, USA).

The potential ecological risk (RI) of rhizospheric soil data was plotted using the Quantum Geographic Information System (QGIS), an open-source desktop application that provides data and allows the user to create maps with different projections. QGIS creates maps composed of raster or vector layers. The distribution maps were performed with QGIS 3.22.4, creating a layer covering the sampled points, on which the interpolation is performed using the inverse distance weighting (IDW) method [56]. 

## 3. Results and Discussion

### 3.1. Behavior of Piptatherum miliaceum 

#### 3.1.1. Rhizospheric Soil Characterization

Native plants were investigated as potential metal(loid) bio-monitors on the dry riverbed in the mining District Cartagena-La Union. The species to be used as a candidate bio-monitor considers multiple factors: (i) it is widespread and abundantly found in the studied area, (ii) it is tolerant to the aridity, salinity, and polluted ecosystem conditions, (iii) it provides sufficient biomass for pollutants analysis, and (v) knowledge on its potential to accumulate metal(loid)s from the study area. When plants are used as bio-monitors for metal(loid)s it is important to take the physical–chemical characteristics of the soil into account [2]. The most dominant plant in the EB dry riverbed was *Piptatherum miliaceum*, where rhizospheric soil had a wide range of pH on the surface (3.51–7.41) (Supplementary Materials). These values were categorized as extremely acidic near the headwater, and slightly alkaline near the coast [40], indicating that it is a species tolerant to different pH conditions. The mean values close to the mouth were like those reported at the mouth of the EB dry riverbed by Conesa et al. [39], who found for rhizospheric soils a pH of 7.13. Overall, electrical conductivity (EC) stayed constant with small variation along the course of the riverbed, with the highest value located close to the headwater (3.21 mS cm^−1^) and the lowest (2.35 mS cm^−1^) in the middle of the riverbed (Supplementary Materials). In addition, results showed that, overall, the dominant cation was Ca^2+^ (3060 mg kg^−1^) with SO_4_^2−^ (8500 mg kg^−1^) as the main anion, (Table 1). In EB the soluble concentration of SO_4_^2−^ exceeded 1440 mg L^−1^, which Alarcón-Vera [57] considered as phytotoxic to plant growth, which confirmed that this species is growing in an adverse environment. Additionally, the rhizospheric soil was categorized as loamy sand, with a mean sand percentage along the surface of 83%, silt of 15%, and clay of 1.2% [40] (Table 1). The OC ranged from 0.14% near the coast to 0.75% close to the headwater. Overall, on the dry riverbeds, the main particle size was sand with differences in the OC content. This variation in OC is one of the main causes of plant colonization [58].

The total metal(loid) mean concentrations ranged as: As (182–426 mg kg^−1^), Cd (1.7–14.3 mg kg^−1^), Cu (52–129 mg kg^−1^), Cr (24.1–34.4 mg kg^−1^), Fe (63,634–117,142 mg kg^−1^), Mn (296–5446 mg kg^−1^), Ni (10.8–24.7 mg kg^−1^), Pb (2054–7778 mg kg^−1^), and Zn (1462–5487 mg kg^−1^) (Supplementary Materials). 

**Figure 2:**
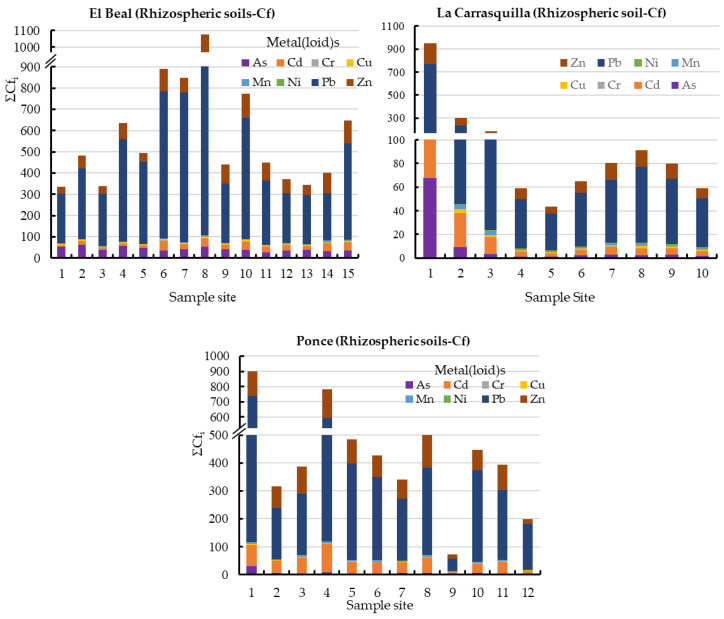
Contamination factor contribution ΣCfi in rhizospheric soil samples from dry riverbeds, El Beal (*Piptatherum miliaceum*); La Carrasquilla (*Foeniculum vulgare*); Ponce (*Dittrichia viscosa*). Arsenic (As), cadmium (Cd), chromium (Cr), copper (Cu), iron (Fe), manganese (Mn), nickel (Ni), lead (Pb), zinc (Zn).

In this study area, all rhizospheric soils sampled exceeded the background values for As, Cd, Cu, Pb, and Zn [47]; there is no background concentration defined for Fe. Those five metals(loid)s showed mean values of Cf along the surface for As (42.2), Cd (24.6), Cu (6.24), Pb (414), and Zn (77.3) (Figure 2). According to those values, in general, the rhizospheric soil was very highly contaminated for those five metals(loid)s (Cf ≥ 6), moderately contaminated for Mn (1 ≤ Cf < 3), and lowly contaminated for Cr and Ni (Cf < 1) [46].

**Figure 3:**
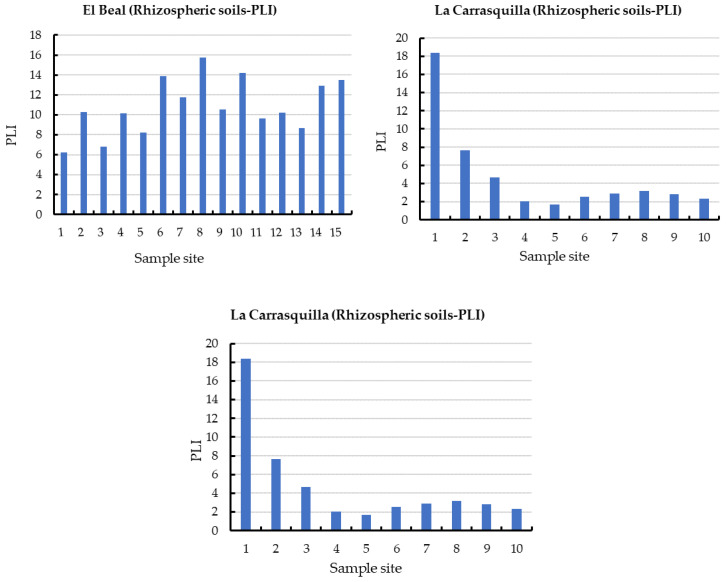
Pollution load index (PLI) in rhizomes samples from dry riverbeds. El Beal (*Piptatherum miliaceum*); La Carrasquilla (*Foeniculum vulgare*); Ponce (*Dittrichia viscosa*). Arsenic (As), cadmium (Cd), chromium (Cr), copper (Cu), iron (Fe), manganese (Mn), nickel (Ni), lead (Pb), zinc (Zn).

In addition, the PLI values along the surface in EB rhizospheric soil were higher than 6.25 (Figure 3), with values of PLI > 1, these values classify the soil as contaminated by metal(loid)s [49]. In addition, the metal(loid)s that represented the highest individual potential risk Eri were Cd (22.1%) and Pb (61.8%) (Supplementary Materials). 

**Figure 4:**
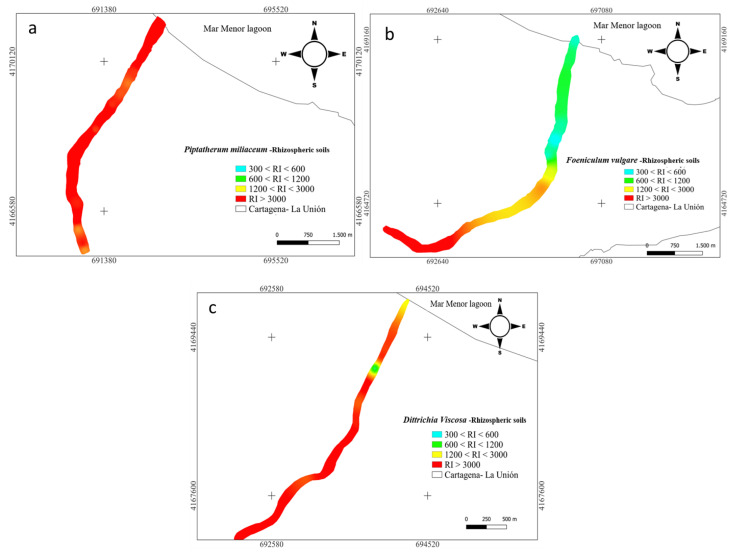
Spatial distribution of risk index RI in rhizome samples from dry riverbeds. (**a**) El Beal (*Piptatherum miliaceum*); (**b**) La Carrasquilla (*Foeniculum vulgare*); (**c**) Ponce (*Dittrichia viscosa*).

Furthermore, for the nine metal(loid)s analyzed (As, Cd, Cr, Cu, Mn, Ni, Pb, and Zn), the RI index categorized all the samples (15) (Figure 4) as significantly high ecological risk (RI ≥ 600) [45] with a mean value along the surface of 3346. Cuevas et al. [24] studied spatial distributions of metal(loid)s in the EB dry riverbed and reported a RI mean value of 3799 for topsoil.

The bioavailable elements in the soil are accessible to the roots and can be accumulated in all parts of the plant [2]. The highest bioavailable metal(loid) fractions compared to total concentration were for Cd (27.2%), Pb (21.5%), and Zn (11.9%), with mean concentrations along the surface of 2.14 mg kg^−1^, 828 mg kg^−1^, and 380 mg kg^−1^, respectively. García et al. [16] reported from the EB dry riverbed Pb contamination for the rhizospheric soils (total Pb 46,000 mg kg^−1^ and bioavailable 557 mg kg^−1^). On the contrary, soluble fractions were close to zero except for Cd, Zn with mean concentrations of 0.71 mg kg^−1^ and 108 mg kg^−1^, respectively. 

#### 3.1.2. Root–Stem Characterization 

In order to consider native plants for use as a bio-monitor, there were parameters established according to the bioaccumulation factor (BF), mobility ratio (MR), and linear correlations between metal(loid) concentrations in plant tissues (root or stem) in rhizospheric soils. Bio-monitor plants must have a linear relationship, rhizospheric soil:root, to establish an unequivocal statement as a contamination monitor [59]. While the BF expresses the efficiency of the plant in accumulating metal(loid)s from soil [51]. Metal(loid) concentrations in roots for the five metal(loid)s that exceeded background values in all rhizospheric soils ranged from 15 to 102 mg kg^−1^ for As, 1.6 to 25.5 mg kg^−1^ for Cd, 16 to 50 mg kg^−1^ for Cu, 262 to 2950 mg kg^−1^ for Pb, and 392 to 2368 mg kg^−1^ for Zn (Supplementary Materials). Phytotoxic concentrations in plants established by Kabata-Pendias [60] are shown in Table 2. Based on the results, there were phytotoxic concentrations in the range or above in all root samples for As, Pb, and Zn. Meanwhile, phytotoxic concentrations on roots for Cd and Cu were 48% and 73% for the sampling points respectively (Supplementary Materials).

**Figure 5:**
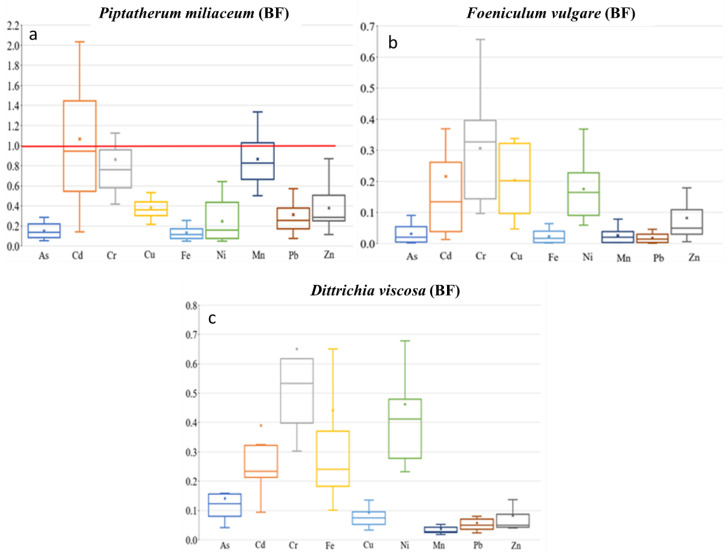
Box plots for bioaccumulation factor (BF) of the 9 metal(loid)s by plant species–dry riverbed. “x” identifies the mean value. (**a**) El Beal (*Piptatherum miliaceum*); (**b**) La Carrasquilla (*Foeniculum vulgare*); (**c**) Ponce (*Dittrichia viscosa*).

*Piptatherum miliaceum* showed similar concentrations along the riverbed for roots and rhizospheric soil with a BF value of 1.07 for Cd, (Figure 5), while the metal(loid)s with the highest concentrations on rhizospheric soil and the root showed lower BF, Pb (0.29), and Zn (0.34). In contrast, using the bioavailable concentration of metals, the index BF_b_ increased for Cd = 4.26, Pb = 1.90, and Zn = 3.91. García et al. [16] studied pots artificially contaminated with Pb and Zn applied as acetate and found that *Piptatherum miliaceum* from the Cartagena-La Unión mining district was more efficient at removing Pb (BF_b_ = 4.17) from the soil than Zn (BF_b_ = 2.85). When certain metals are present, a reciprocal inhibition of heavy metal uptake can occur, and has been observed in plants grown in the presence of Zn and other metals [61]. Additionally, Ebbs et al. [62] found that interactions between metals can decrease the uptake of one metal in the presence of others. 

Mean metal(loid) concentration presented *Piptatherum miliaceum* from the EB dry riverbed as a metal(loid) monitor for Cd with similar means concentrations on rhizospheric soil and roots (Table 2). Spearman correlation analysis showed a positive and significant correlation between rhizospheric soil–root metal(loid) concentration for Cd (r = 0.61, *p* = 0.02) and Mn (r = 0.61, *p* = 0.02), however, Mn showed a low BF (0.25). Additionally, there was a positive correlation between Cd for rhizospheric soil bioavailable fraction and root concentration (r = 0.51, *p* = 0.05) (Supplementary Materials). 

*Piptatherum miliaceum* presented for the metal(loid)s with values above the background in all the rhizospheric soils a stem concentration lower than the root for As (1.75–17.5 mg kg^−1^), Cd (0.23–12.8 mg kg^−1^), Cu (6.02–24.0 mg kg^−1^), Pb (51.8–623 mg kg^−1^), and Zn (148–1116 mg kg^−1^) (Supplementary Materials). In the EB dry riverbed, there were phytotoxic concentrations in all the stems sampled for Pb and Zn, while for As, Cd, and Cu there were phytotoxic concentrations in 73%, 27%, and 13% of the sampled plants, respectively (Supplementary Materials).

**Figure 6:**
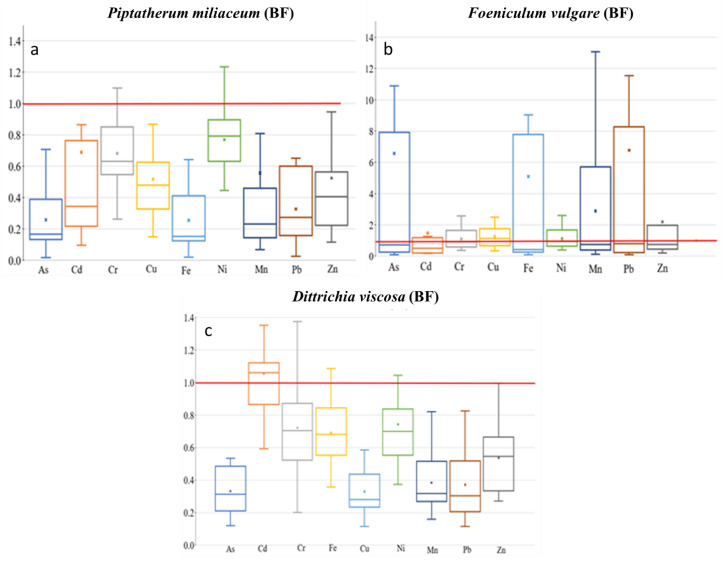
Box plots for translocation factor (TF) of the 9 metal(loid)s by plant species–dry riverbed. “x” identifies the mean value (**a**) El Beal (*Piptatherum miliaceum*); (**b**) La Carrasquilla (*Foeniculum vulgare*); (**c**) Ponce (*Dittrichia viscosa*).

**Figure 7:**
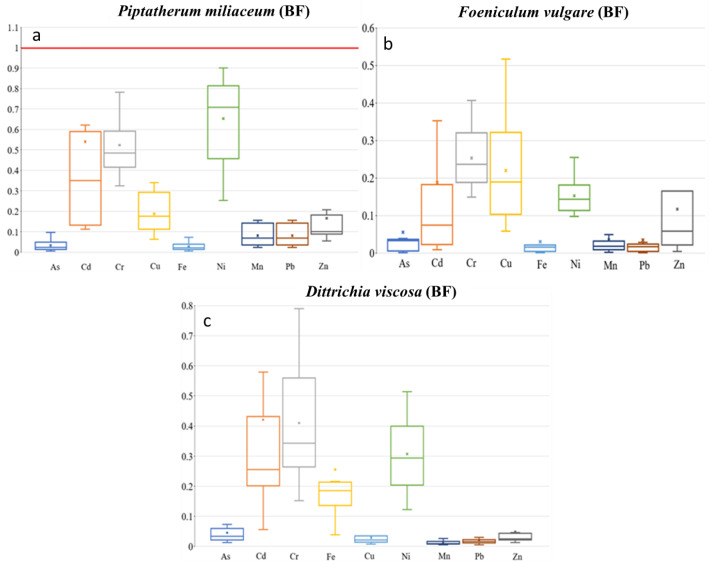
Box plots for mobility ratio (MR) of the 9 metal(loid)s by plant species–dry riverbed. “x” identifies the mean value (**a**) El Beal (*Piptatherum miliaceum*); (**b**) La Carrasquilla (*Foeniculum vulgare*); (**c**) Ponce (*Dittrichia viscosa*).

The TF showed the highest values on Cd (0.69), among As, Cd, Cu, Pb, and Zn (Figure 6), with a moderate–no significant correlation between root and stem metal(loid) concentration for Cd (r = 0.50, *p* = 0.06). In addition, the MR showed the highest value on Cd (0.54), with no significant correlation between stem–rhizospheric total concentration. The metal(loid)s with a higher concentration on the stem presented lower MR, Pb (0.08), and Zn (0.17) (Figure 7). The ratio stem:bioavailable rhizospheric concentrations showed MR_b_ on Cd (2.20), Pb (0.43), and Zn (1.75), with no significant correlation between them. Those MR_b_ values were lower than the ratio reported by García et al. [16] (Pb = 0.95, Zn = 6.18), who used a bioavailable combination of 900 mg Pb kg^−1^ and 300 mg Zn kg^−1^. 

*Piptatherum miliaceum* showed characteristics that can be considered as a potential bio-monitor for Cd, having a strong correlation root–rhizospheric soil, r > 0.61, and a BF > 1. In general, *Piptatherum miliaceum* (EB) showed a decreasing trend in metal(loid) concentration rhizospheric soil > roots > stem, except for Cd, with a mean value 6.86% higher on roots. It must be considered that Cd is very toxic for any kind of organism, and as far as is known, Cd does not constitute a metabolically important compound [63]. Conesa et al. [64], who studied the uptake of *Piptatherum miliaceum* for different metal concentrations, reported higher concentrations on roots for Cd, Cu, Pb, and Zn than soil concentrations. In addition, a study of different Mediterranean plants used for phytoremediation of mine soils polluted by cadmium, which was carried out in a tailing pond in the Cartagena-La Unión Mining District, reported a BF = 0.95 for Cd on *Piptaterum miliaceum* and pointed out that the plant accumulated higher quantities of Cd in the roots than in the shoots [65]. However, previous studies have reported the absence of correlations between Cd in plant tissues and Cd in soil [23,66]. Differences among studies could be due to different climate conditions and soil characteristics, such as nutrient levels, organic matter, Cd speciation, and pH.

### 3.2. Behavior of Foeniculum vulgare 

#### 3.2.1. Rhizospheric Soil Characterization 

Environmental gradients, including salinity, redox conditions, soil moisture, and pH, determine the plant distribution [67,68]. *Foeniculum vulgare* was the dominating plant in the LC dry riverbed, the rhizospheric soil pH presented small variation along the surface (7.62–8.5), and it was classed between slightly alkaline and moderately alkaline [40]. According to Gonzales-Fernandez et al. [21], metals are readily disseminated to various environmental compartments, including soils and biota, due to their high solubility at low pH. In the case of electrical conductivity (EC), except for sample (1) in the headwaters (0.003 mS cm^−1^), there were small variations along the riverbed (Supplementary Materials), with a mean value of 0.30 mS cm^−1^. The predominate cation was Ca^2+^ (581 mg kg^−1^), while SO_4_^2−^ (1329 mg kg^−1^) was the main anion. On the other hand, a sandy loam classification was given to the rhizospheric soil, with a mean sand content of 62%, silt 34%, and clay 3.5% (Table 1). The OC varied from 0.05% near the headwater to 1.91% on half of the surface. Cuevas et al. [24] reported for the LC dry riverbed topsoil along the surface mean values for pH (7.94), EC (2.43 mS cm^−1^), and for the main ions with mean values of Ca^2+^ (1277 mg kg^−1^) and SO_4_^2−^ (4061 mg kg^−1^).

In the rhizosphere soil of *Foeniculum vulgare*, metal(loid) total concentrations varied from 7.07 to 475 mg kg^−1^ for As, 0.83 to 26.1 mg kg^−1^ for Cd, 10.8 to 17.5 mg kg^−1^ for Cr, 12.1 to 74.0 mg kg^−1^ for Cu, 8708 to 142,403 mg kg^−1^ for Fe, 719 to 7377 mg kg^−1^ for Mn, 12.4 to 19.1 mg kg^−1^ for Ni, 290 to 5599 mg kg^−1^ for Pb, and 233 to 7502 mg kg^−1^ for Zn (Supplementary Materials). According to background levels proposed by Martinez Sánchez et al. [47], all samples had concentrations above the backgrounds for As, Cd, Pb, and Zn. The mean Cfs for those metal(loid)s were As (9.44), Cd (15.6), Pb (125), and Zn (36.1), respectively (Figure 2). However, from all the sampling points only Pb (31.2) and Zn (6.00) presented values categorized as very highly contaminated (Cf ≥ 6). Rhizospheric soil had moderate contaminations for Cu and Mn (1 ≤ Cf < 3), and low contamination for Cr and Ni (Cf < 1) [46]. According to the PLI index, along the riverbed in *Foeniculum vulgare* rhizospheric soil was higher than 1.70, which classified all the samples as contaminated by metal(loid)s (Figure 3). Additionally, the metal(loid)s with the highest individual potential ecological risks (E_ri_) were 37.8% for Cd and 50.3% for Pb (Supplementary Materials). Also, the potential ecological risk showed values with considerable ecological risk, RI ≥ 600 in the headwater, while 60% showed values categorized with high ecological risk, 300 ≤ RI < 600 (Figure 4). 

The highest ratios between bioavailable:total concentrations were for Cd (19.3%), Pb (8.08%), and Zn (6.05%), with mean concentrations along the riverbed of 0.96, 93.8, and 90.4 mg kg^−1^ respectively. The soluble fractions were close to zero for the nine metal(loid)s examined (Table 3). Cuevas et al. [24] studied the LC dry riverbed and reported the bioavailable concentration in the topsoil with respect to the total concentration of Cd (14.5%), Pb (5.15%), and Zn (5.00%). 

#### 3.2.2. Root–Stem Characterization 

*Foeniculum vulgare* roots had metal(loid) concentrations ranging as: As (0.04–1.26 mg kg^−1^), Cd (0.06–0.79 mg kg^−1^), Pb (0.77–24.9 mg kg^−1^), and Zn (16.6–76.5 mg kg^−1^), which corresponded to metal(loid)s that had the highest Cf in the LC dry riverbed (Figure 2). The metal(loid) concentration in *Foeniculum vulgare* roots did not show phytotoxic classification for those four metals(loid)s (As, Cd, Pb, and Zn) (Table 2). These could be attributed to the overall low bioavailable and soluble concentrations (Table 3). In addition, even though all sampling points presented PL1 > 1, 60% showed PLI < 3 (Figure 3). Soil properties could affect metal distribution, mobility, bioavailability, and toxicity [69].

*Foeniculum vulgare* showed the highest BF values for Cd (0.22) among the four metal(loid)s that exceeded the background values in all sampling points, As (0.03), Pb (0.02), and Zn (0.08) (Figure 5). There were no BF values above 0.3 for the nine metal(loid)s analyzed. In addition, *Foeniculum vulgare* metal(loid) concentrations on rhizospheric soil and roots were not similar on any of the nine elements analyzed, with no significant correlation (*p* > 0.05) (Supplementary Materials). Conesa et al. [39] studied *Foeniculum vulgare* from the EB dry riverbed, indicating a BF for Cd of 0.20. There was only a moderate–no significant correlation between rhizospheric soil and root concentration on As (r = 0.50, *p* = 0.14). The BF index for the bioavailable concentrations (BF_b_) were: As (16.6), Cd (0.98), Pb (0.13), and Zn (1.25). Those values were similar to the values found by Conesa et al. [39] of As (19.1), Cd (0.89), Pb (0.14), and Zn (0.20). The strongest correlation between root concentration and the rhizospheric soil bioavailable fraction was for Zn (r = 0.43, *p* = 0.21). 

The concentrations of metal(loid)s in the stems varied from 0.10 to 6.56 mg kg^−1^ for As, 0.05 to 0.89 mg kg^−1^ for Cd, 1.78 to 114 mg kg^−1^ for Pb, and 13.9 to 228 mg kg^−1^ for Zn. There were phytotoxic concentrations on the stems in only one sample point for As (6.56 mg kg^−1^), Pb (114 mg kg^−1^), and Zn (227 mg kg^−1^) (Supplementary Materials). The mobility ratio (MR) stem:rhizospheric soil concentration presented the highest values on Cd (0.19), and any of the other eight metal(loid)s surpassed 0.25 for MR (Figure 7). In addition, the Spearman correlation showed a negative or no significant association between the metal(loid)s. The mean MR_b_ values in the LC *Foeniculum vulgare* were Cd = 0.76, Pb = 0.19, and Zn = 1.63. There were no correlations between concentration on the stem and rhizospheric bioavailable fraction (Supplementary Materials). The MR_b_ in the EB *Foeniculum vulgare* reported by Conesa et al. [39] showed Cd = 1.04, Pb = 0.01, and Zn = 0.21. 

According to Mykolenko et al. [17], *Foeniculum vulgare* is appointed as a strong phytoextractor for As, Cu, Ni, Pb, and Zn due to its translocation factor (TF). The TFs on *Foeniculum vulgare* along the riverbed for metal(loid)s were As (6.57), Cd (1.47), Pb (6.77), and Zn (2.19) (Figure 6). A similar value was reported from the EB dry riverbed for TF-Cd (1.16) by Conesa et al. [39]. Furthermore, in the LC *Foeniculum vulgare*, five of the other elements presented TF > 1, As (6.57), Fe (5.10), Mn (2.89), Pb (6.77), and Zn (2.19). In addition, there were negative or no significant correlations between root and stem metal(loid) concentrations (Supplementary Materials). 

### 3.3. Behavior of Dittrichia viscosa

#### 3.3.1. Rhizospheric Soil Characterization 

The study on the PN dry riverbed showed that the most common plant along that surface was *Dittrichia viscosa*, and the pH of the rhizosphere varied from 6.90 to 8.19, classified as neutral and moderately alkaline, respectively (Supplementary Materials). The EC values along the surface were generally low, categorized as not saline 0–2 mS cm^−1^ [40]. Sample point 1 was the highest value (1.00 mS cm^−1^) located close to the headwater, while the value along the riverbed was around 0.29 mS cm^−1^ (Table 1). The water-soluble ion extraction pointed out that the dominant cation was Na^+^ (510 mg kg^−1^), and NO_3_^−^ (878 mg kg^−1^) was the main anion. On the other hand, this rhizosphere soil was mainly composed of sand, with a mean percentage of 67%, silt 30%, and clay 3.0%. In the middle of the riverbed, the OC was 1.71% and varied to 0.41% close to the coast (Supplementary Materials). 

Metal(loid) total concentrations ranged as: As (5.46–206 mg kg^−1^), Cd (2.67–32.7 mg kg^−1^), Cr (8.97–18.7 mg kg^−1^), Cu (7.61–73.9 mg kg^−1^), Fe (6715–62,239 mg kg^−1^), Mn (1131–4228 mg kg^−1^), Ni (7.29–20.6 mg kg^−1^) Pb (419–5761 mg kg^−1^), and Zn (595–7750 mg kg^−1^). All samples have exceeded the background values for Cd, Mn, Pb, and Zn, while for As only one sample point (9) did not surpass the background level [47]. The mean contamination factor (Cf) on rhizospheric soil was very highly contaminated (Cf ≥ 6) for As (6.77), Cd (45.5), Pb (289), and Zn (89.9). In addition, Cd, Pb, and Zn were metal(loid)s with Cf ≥ 6 in all sample points. Additionally, the PLI values along the riverbed in *Dittrichia viscosa* rhizospheric soil were higher than 2.18 (Figure 3), which classified all samples as contaminated by metal(loid)s. The metal(loid)s that represented the highest potential risks (E_ri_) were Cd (46%) and Pb (48%) (Supplementary Materials). The ecological risk index (RI) showed (Figure 4) an overall significantly high ecological risk (RI ≥ 600) with a mean value of 2985. 

The highest ratio between bioavailable:total concentrations were for Cd (18.4%), Pb (7.29%), and Zn (6.36%), with mean concentrations along the surface 2.67 mg kg^−1^, 196 mg kg^−1^, and 237 mg kg^−1^ respectively (Table 3). Soluble fractions were close to zero for the nine metal(loid)s analyzed. Cuevas et al. [24] reported from PN topsoil a ratio bioavailable:total concentration for Cd (14.5%), Pb (6.8%), and Zn (5.5%). 

#### 3.3.2. Root–Stem Characterization 

*Dittrichi viscosa* is characterized to tolerate and accumulate high concentrations of metals (~2000 mg Pb kg^−1^) in its roots [70]. Metal(loid) concentrations in *Dittrichia viscosa* roots ranged as: As (1.60–8.46 mg kg^−1^), Cd (0.44–14.2 mg kg^−1^), Pb (27.7–277 mg kg^−1^), and Zn (81.6–313 mg kg^−1^), which corresponded to a very highly contaminated rhizospheric soil (Cf ≥ 6). There were phytotoxic concentrations on roots for Pb and Zn, except at sample point 9 (Table 2). The ratio of means metal(loid) concentrations in roots against the minimum phytotoxic concertation for a single metal(loid)s showed the highest values for Pb (4.72) and Zn (2.13). 

*Dittrichia viscosa* from the PN dry riverbed showed BF values for As (0.14), Cd (0.39), Pb (0.06), and Zn (0.08) (Figure 5); values below 0.2 are considered normal when plants are grown on polluted materials [71]. Any of the other metal(loid)s presented a BF higher than 0.65. On the other hand, the study in the mouth of EB dry riverbed—*Dittrichia viscosa* by Conesa et al. [64] found the highest BF values for Cd = 0.15, with lower values for Pb = 0.01 and Zn = 0.02 There were positive moderate correlations between rhizospheric soil and root concentration (Supplementary Materials). However, correlation analysis between them presented a significant (*p* < 0.05) correlation only with As. The positive correlations between them were: As (r = 0.57, *p* = 0.05), Cd (r = 0.43, *p* = 0.17), Pb (r = 0.47, *p* = 0.12), and Zn (r = 0.55, *p* = 0.06). According to Nogales and Benítez [72], during a study in pots adding a saturated aqueous solution of Pb (3200 mg kg^−1^) in the form of PbCl_2_ and Zn (4000 mg kg^−1^) in the form of ZnCl_2_, showed Pb and Zn concentrations in *Dittrichia viscosa* noticeably higher in roots, with BF values for Pb (0.99) and Zn (0.41). Bioaccumulation of metals by plants is affected by plant species, physiological adaptation, plants growing stage, and element characteristics that control absorption [73].

The BF_b_ in the PN *Dittrichia viscosa* presented values of 0.71 for Cd, 1.22 for Pb, and 3.45 for Zn (Supplementary Materials). There was a moderate positive and significant correlation between root concentration and rhizospheric soil bioavailable fraction on Pb (r = 0.64, *p* = 0.02), also there were moderate–no significant correlations on Zn (r = 0.50, *p* = 0.10). Nogales and Benítez [72] reported BF_b_ for Pb (9.96) and Zn (1.31). 

Metal(loid) concentrations in the stem ranged as: Cd (0.48–11.6 mg kg^−1^), As (0.60–2.56 mg kg^−1^), Pb (16.5–75.9 mg kg^−1^), and Zn (72.8–151 mg kg^−1^). In general, *Dittrichia viscosa* presented the highest BF and MR values near the coast for the nine metal(loid)s. The mobility ratio represented the ratio stem:rhizospheric soil concentration with values on Cd (0.30), Pb (0.05), and Zn (0.01) (Figure 7). Additionally, there were moderate–no significant correlations between shoot–rhizospheric soil on Pb (r = 0.51, *p* = 0.09) (Supplementary Materials). Nogales and Benítez [72] found values for Pb (0.00) and Zn (0.05), while Conesa et al. [39] reported values for Pb (0.03) and Zn (0.09).

Gonzales-Fernandez et al. [21] reported, for *Dittrichia viscosa* growing in the mine waste area, using an energy dispersive X-ray fluorescence (EDXRF), a TF value for Zn of 2.47. However, in the PN dry riverbed the plant showed lower TF values for Zn (0.38) (Figure 6). 

## 4. Conclusions

*Piptatherum miliaceum*, *Foeniculum vulgare*, and *Dittrichia viscosa* exhibited a good adaptation to heavily polluted soil, this being the case in more than 50% of the sample points established with a good develop of biomass. These plants showed resilience to soil with extremely acid pH (*Piptaterum miliaceum*), low organic matter, and with a significantly high ecological risk. The three studied dry riverbeds, El Beal, La Carrasquilla, and Ponce, showed metal(loid) contamination in the rhizospheric soil, with a pollution index above 1. The results demonstrate that the metal(loid) concentrations exceeded the normal range for As, Cd, Cu, Mn, Pb, and Zn. *Piptatherum miliaceum* from El Beal demonstrated potential as a bio-monitor for Cd. This is due to its correlation in the Cd concentration between roots and rhizospheric soil, and a bioaccumulation factor exceeding 1. Overall, *Piptatherum miliaceum* had a decreasing sequence in metal(loid) concentration from rhizospheric soil to roots to stem, with the exception of Cd, which had a higher mean concentration in the roots. *Foeniculum vulgare* and *Dittrichia viscosa* showed high BF, MR for Cr, but they never reached values of BF > 0.60. Additionally, Cr concentrations were under the background levels, considered as lithogenic concentration. 

## Figures and Tables

**Table 1:** Geochemical properties of rhizospheric soil (mean ± standard deviation) (n = 3).

Rhizospheric Soil	*P. milleaceum* El Beal (n = 15)	*F. vulgare* La Carrasquilla (n = 10)	*D. viscosa* Ponce (n = 12)
pH	5.14(1.10)	8.24(0.25)	7.82(0.32)
EC (mS cm^−1^)	2.56(0.22)	0.30(0.13)	0.29(0.24)
OC (%)	0.42(0.17)	13.5(5.42)	0.92(0.40)
IC (%)	0.17(0.15)	1.84(0.74)	1.86(1.41)
Clay (%)	1.26(0.87)	3.52(1.79)	2.88(1.46)
Silt (%)	15.3(4.61)	34.2(14.9)	29.1(11.7)
Sand (%)	83.5(5.45)	62.3(16.7)	68.1(13.1)
Ca^2+^ (mg kg^−1^)	3060(140)	582(1149)	41.4(25.3)
Mg^2+^ (mg kg^−1^)	300(224)	190(461)	199(0.93)
Na^+^ (mg kg^−1^)	90.6(52.4)	260(691)	510(894)
Cl^−^ (mg kg^−1^)	76.3(74.6)	420(1215)	12.7(5.66)
NO_3_^−^ (mg kg^−1^)	6020(1642)	88.5(218)	878(2108)
SO_4_^2−^ (mg kg^−1^)	8500(864)	1329(3666)	54.8(16.2)

EC: electrical conductivity; OC: organic carbon; IC: inorganic carbon Ca^2+^: calcium; Mg^2+^: magnesium; Na^+^: sodium; Cl^−^: chloride; NO_3_^−^: nitrate; SO_4_^2−^: sulfate.

**Table 2:** Summary of mean metal(loid) concentrations in rhizospheric soil and plants (mg kg^−1^).

	Phytotoxic *	Rhizospheric Soil	Roots	Stems
*Piptatherum miliaceum*				
As	5.00–20.0	296(63.7)	43.1(24.6)	8.61(5.06)
Cd	5.00–30.0	7.87(3.97)	8.41(7.70)	3.72(3.56)
Cr	5.00–30.0	28.1(3.1)	24.1(10.9)	14.5(2.9)
Cu	20.0–100	78.6(19.7)	29.6(10.4)	14.0(5.9)
Fe	-	87,044(14,584)	11,290(5886)	2325(1511)
Mn	400–1000	2104(1303)	454(453)	96.2(65.2)
Ni	10.0–100	15.2(3.5)	12.6(2.9)	9.38(1.94)
Pb	30.0–300	3849(1878)	1105(703)	284(172)
Zn	100–400	3198(1259)	1089(555)	432(244)
*Foeniculum vulgare*				
As	5.00–20.0	66.1(144.6)	0.61(0.45)	1.00(1.96)
Cd	5.00–30.0	5.00(7.81)	0.37(0.24)	0.25(0.27)
Cr	5.00–30.0	13.8(2.0)	4.15(2.45)	3.41(1.08)
Cu	20.0–100	24.6(19.6)	3.68(1.24)	4.01(1.67)
Fe	-	31,999(39,202)	435(323)	540(950)
Mn	400–1000	1916(2051)	31.9(23.3)	37.3(52.7)
Ni	10.0–100	16.1(2.2)	2.76(1.44)	2.40(0.73)
Pb	30.0–300	1160(1622)	12.2(9.2)	18.7(34.0)
Zn	100–400	1496(2192)	46.1(22.5)	55.4(62.9)
*Dittrichia viscosa*				
As	5.00–20.0	47.4(51.5)	4.51(2.06)	1.33(0.59)
Cd	5.00–30.0	14.6(8.2)	4.36(3.40)	4.36(2.89)
Cr	5.00–30.0	15.3(3.3)	9.18(4.28)	5.79(1.76)
Cu	20.0–100	28.9(16.6)	8.15(5.24)	5.04(1.58)
Fe	-	23,099(14,390)	1592(499)	484(5)
Mn	400–1000	2876(1069)	89.3(26.3)	31.8(10.4)
Ni	10.0–100	16.0(4.0)	6.76(2.65)	4.57(1.20)
Pb	30.0–300	2686(1389)	142(83)	41.0(17.1)
Zn	100–400	3720(2085)	213(68)	104(30)

(*) Phytotoxic plant concentration by Kabata-Pendias [60].

**Table 3:** Summary of mean (n = 3) bioavailable and soluble metal(loid)s in rhizospheric soil (mg kg^−1^).

El Beal rhizospheric soil (*Piptatherum miliaceum*)								
Mean bioavailable metal(loid) concentration (mg kg^−1^)								
**As**	**Cd**	**Cr**	**Cu**	**Fe**	**Mn**	**Ni**	**Pb**	**Zn**
0.06(0.06)	2.14(1.41)	0.02(0.01)	3.56(1.66)	74.3(64.8)	92.1(72.2)	0.38(0.30)	828(525)	380(242)
Mean soluble metal(loid) concentration (mg kg^−1^)								
**As**	**Cd**	**Cr**	**Cu**	**Fe**	**Mn**	**Ni**	**Pb**	**Zn**
0.01(0.01)	0.71(0.60)	0.00(0.00)	0.24(0.50)	0.15(0.33)	37.4(62.4)	0.13(0.11)	3.11(3.90)	108(90.9)
La Carrasquilla rhizospheric soil (*Foeniculum vulgare*)								
Mean bioavailable metal(loid) concentration (mg kg^−1^)								
**As**	**Cd**	**Cr**	**Cu**	**Fe**	**Mn**	**Ni**	**Pb**	**Zn**
0.04(0.02)	0.96(1.52)	0.00(0.00)	1.78(0.89)	9.09(7.16)	39.0(15.6)	0.36(0.14)	93.8(25.4)	90.4(132.5)
Mean soluble metal(loid) concentration (mg kg^−1^)								
**As**	**Cd**	**Cr**	**Cu**	**Fe**	**Mn**	**Ni**	**Pb**	**Zn**
0.03(0.01)	0.00(0.01)	0.00(0.00)	0.04(0.01)	0.45(0.71)	0.07(0.11)	0.01(0.01)	0.00(0.01)	0.09(0.21)
Ponce rhizospheric soil (*Dittrichia viscosa*)								
Mean bioavailable metal(loid) concentration (mg kg^−1^)								
**As**	**Cd**	**Cr**	**Cu**	**Fe**	**Mn**	**Ni**	**Pb**	**Zn**
0.04(0.02)	2.67(1.60)	0.01(0.00)	1.60(0.48)	4.88(1.44)	28.0(9.5)	0.16(0.08)	196(76)	237(125)
Mean soluble metal(loid) concentration (mg kg^−1^)								
**As**	**Cd**	**Cr**	**Cu**	**Fe**	**Mn**	**Ni**	**Pb**	**Zn**
0.02(0.01)	0.01(0.02)	0.00(0.00)	0.05(0.02)	0.24(0.27)	0.27(0.56)	0.01(0.00)	0.04(0.07)	0.28(0.33)

## Data Availability

All data are available in the work/Supplementary Materials referenced.

## Supplementary Materials

The following supporting information can be downloaded at: https://www.mdpi.com/article/10.3390/plants12213775/s1, Metal(loid) concentration (As, Cd, Cr, Cu, Fe, Mn, Ni, Pb, and Zn) on rhizospheric soils, roots, and stems. Geochemical properties of rhizospheric soil from El Beal (*Piptatherum miliaceum*, 15 sampling points), La Carrasquilla (*Foeniculum vulgare*, 10), and Ponce (*Dittrichia viscosa*,) dry riverbeds. Correlation between total metal(loid) concentration in rhizospheric soil and metal(loid) concentration in the root (El Beal (*Piptatherum miliaceum*), La Carrasquilla (*Foeniculum vulgare*), and Ponce (*Dittrichia viscosa*). Correlation between bioavailable metal(loid) concentration in rhizospheric soil and metal(loid) concentration in the root. Correlation between total metal(loid) concentration in rhizospheric soil and metal(loid) concentration in the stem. Correlation between bioavailable metal(loid) concentration in rhizospheric soil and metal(loid) concentration in the stem. Correlation between root metal(loid) concentration and metal(loid) concentration in the stem. Mean values of individual potential ecological risk Eri and total ecological risk RI (rhizospheric total concentrations) for the dry riverbeds. Mean values of bioaccumulation factor BF_b_ (rhizospheric bioavailable concentrations) for the dry riverbeds. Mean values of mobility ratio MR_b_ (rhizospheric bioavailable concentrations) for the dry riverbeds.
