# Supplementary material for: Bio-Monitoring of Metal(loid)s Pollution in Dry Riverbeds Affected by Mining Activity
Source: Plants (Basel). 2023 Nov 5;12(21):3775. doi: 10.3390/plants12213775 (PMC10650150; doi:10.3390/plants12213775)
Supplement: Supplementary file 1 [file plants-12-03775-s001.zip › plants-2652861-supplementary.pdf]

Bio-Monitoring of Metal(loid)s Pollution in Dry Riverbeds Affected by Mining Activity

José Cuevas, Ángel Faz, Silvia Martínez-Martínez, María Gabarrón, Juan Beltrá, Jacinto Martínez and José A. Acosta *

Sustainable Use, Management and Reclamation of Soil and Water Research Group, Universidad Politécnica de Cartagena, Paseo Alfonso XIII 48, 30203 Cartagena, Spain; jose.cuevas@upct.es (J.C.); angel.fazcano@upct.es (Á.F.); silvia.martinez@upct.es (S.M.-M.); m.gabarron@upct.es (M.G.); juan.beltra@upct.es (J.B.); jacinto.martinez@upct.es (J.M.)

* Correspondence: jose.cuevas@upct.es

Supplementary Material

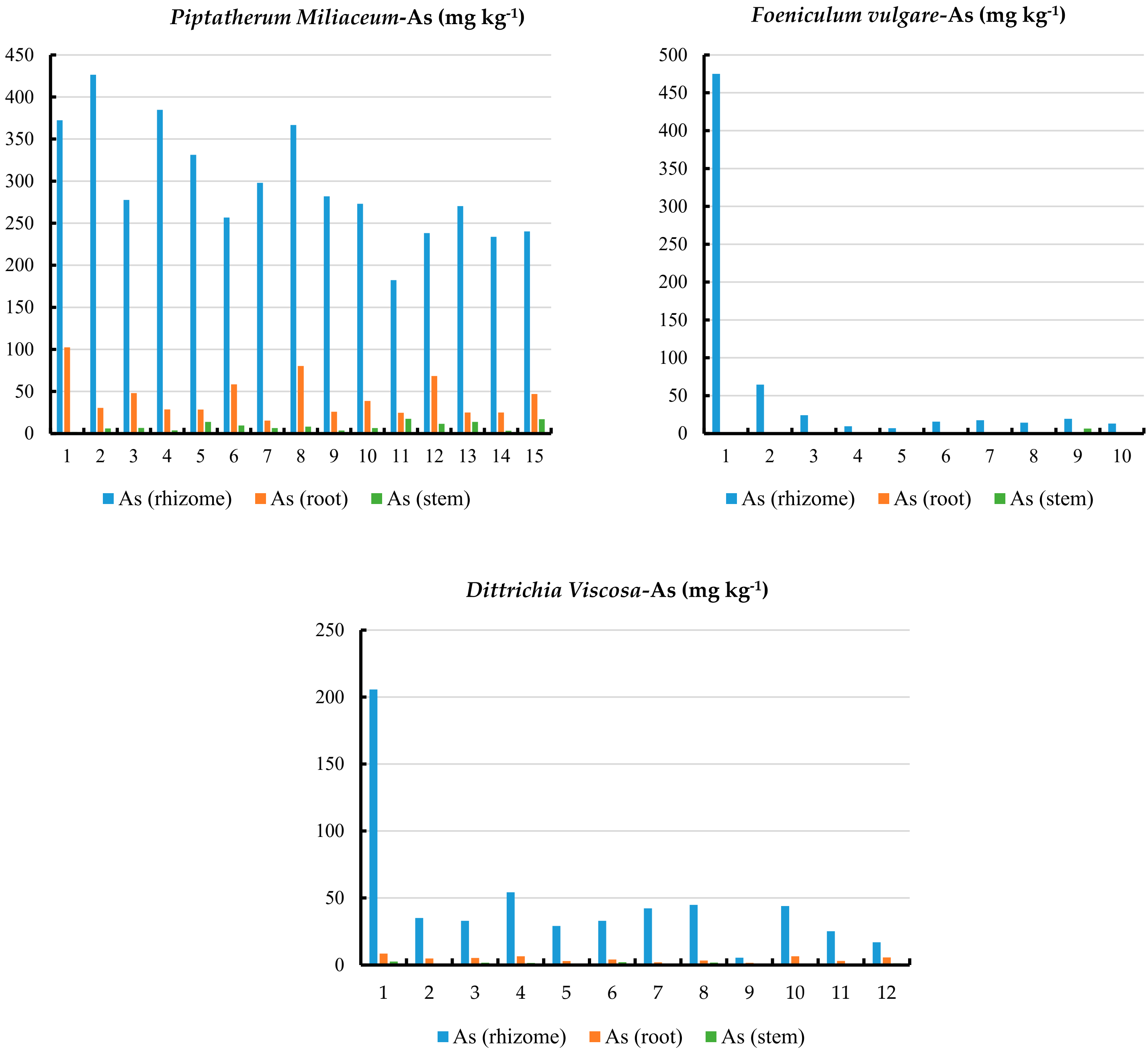

Figure S1. As concentration on sample points.

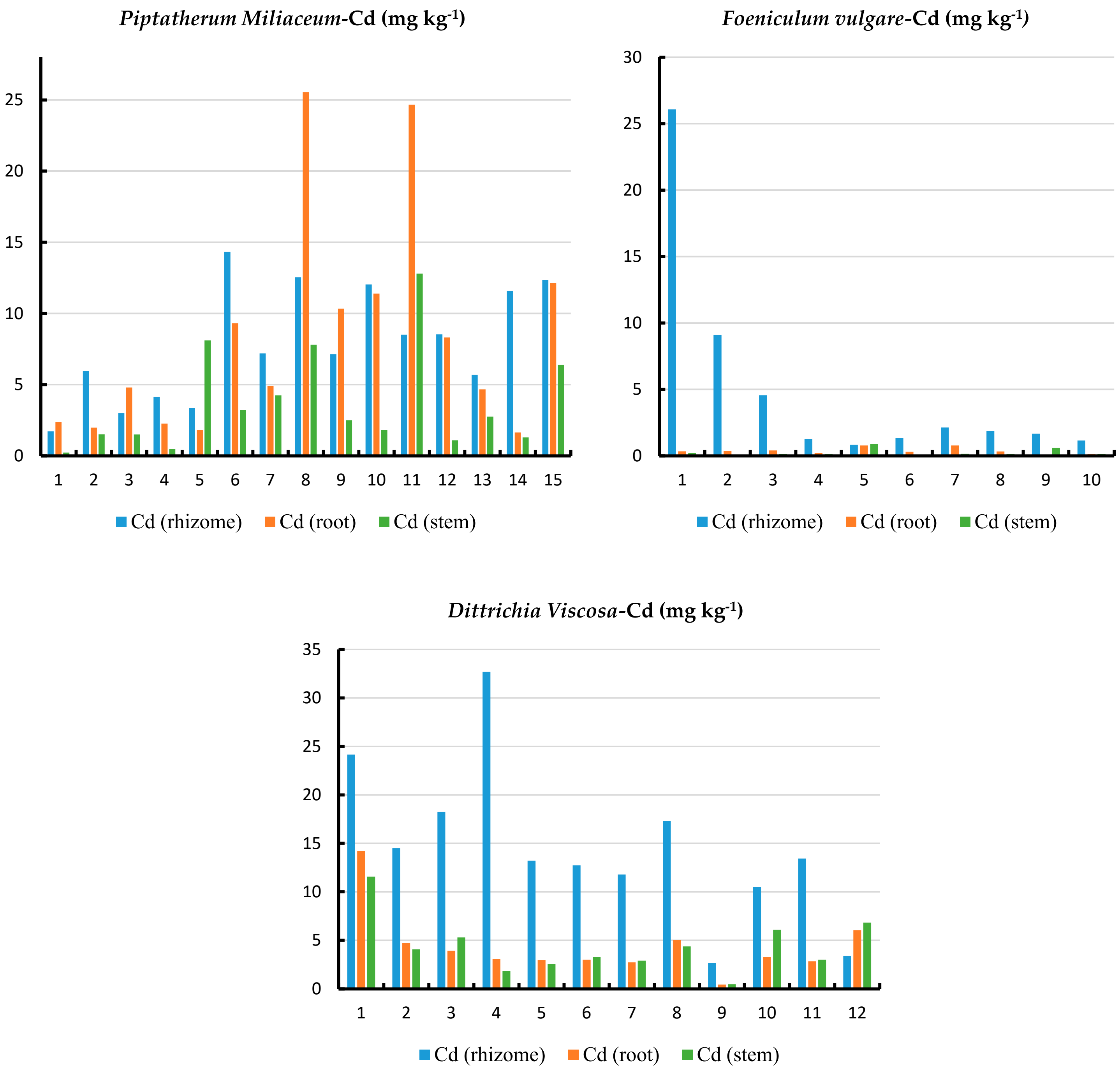

Figure S2. Cd concentration on sample points.

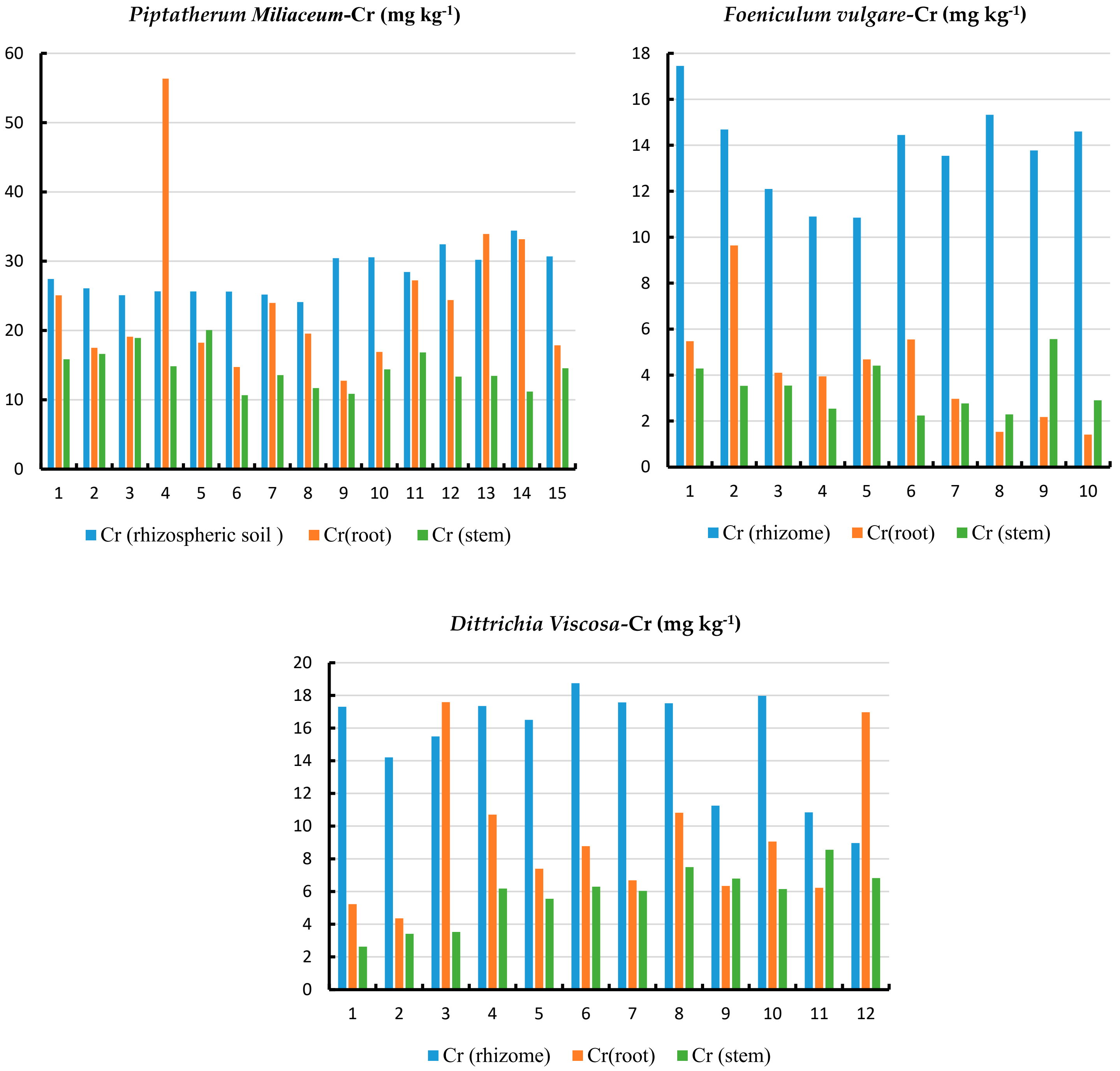

Figure S3. Cr concentration on sample points.

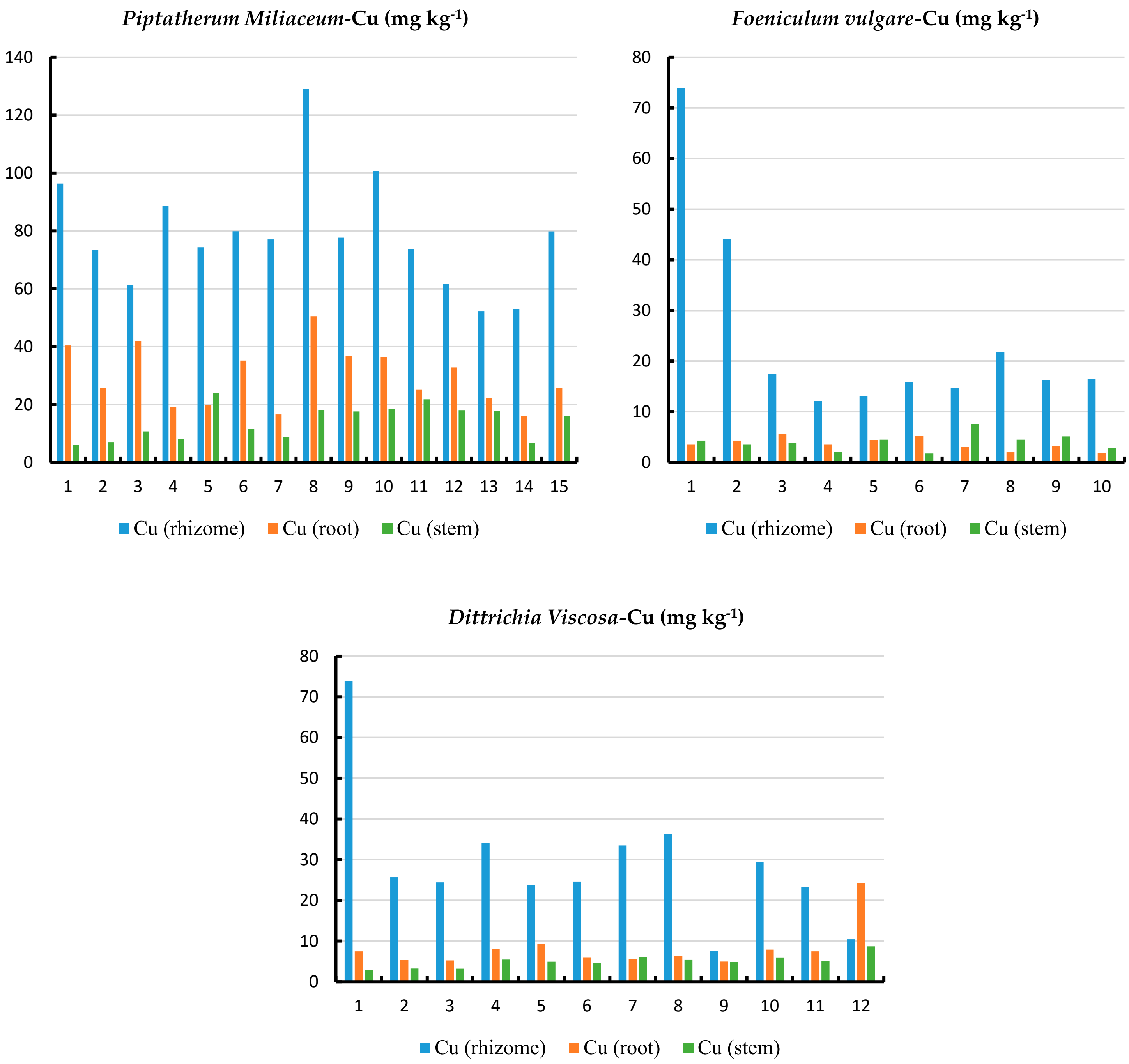

Figure S4. Cu concentration on sample points.

.

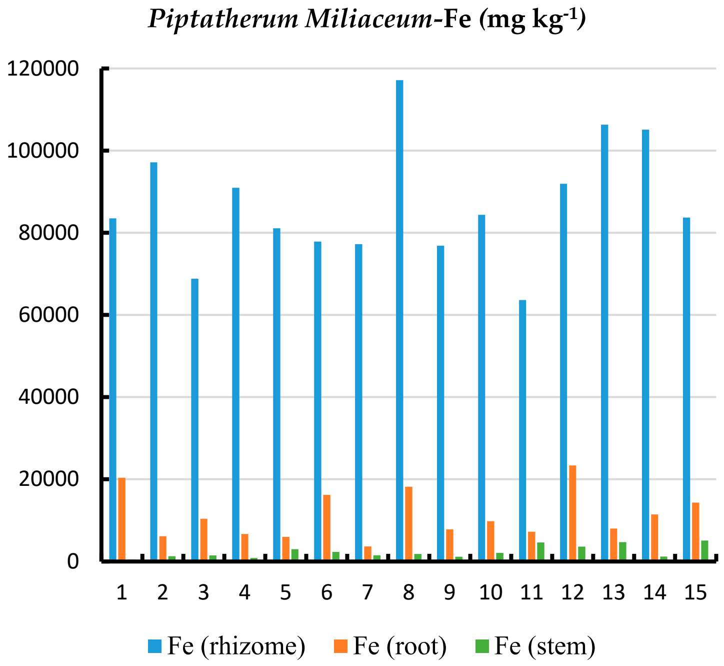

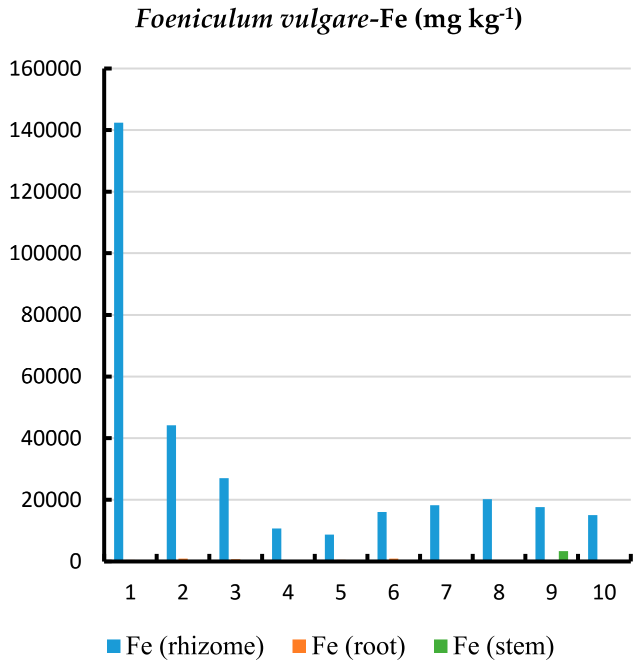

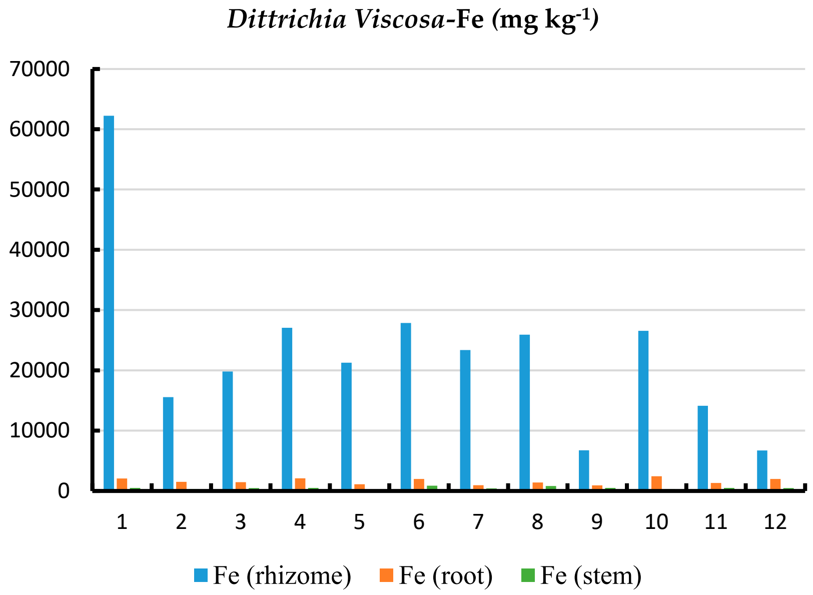

Figure S5. Fe concentration on sample points.

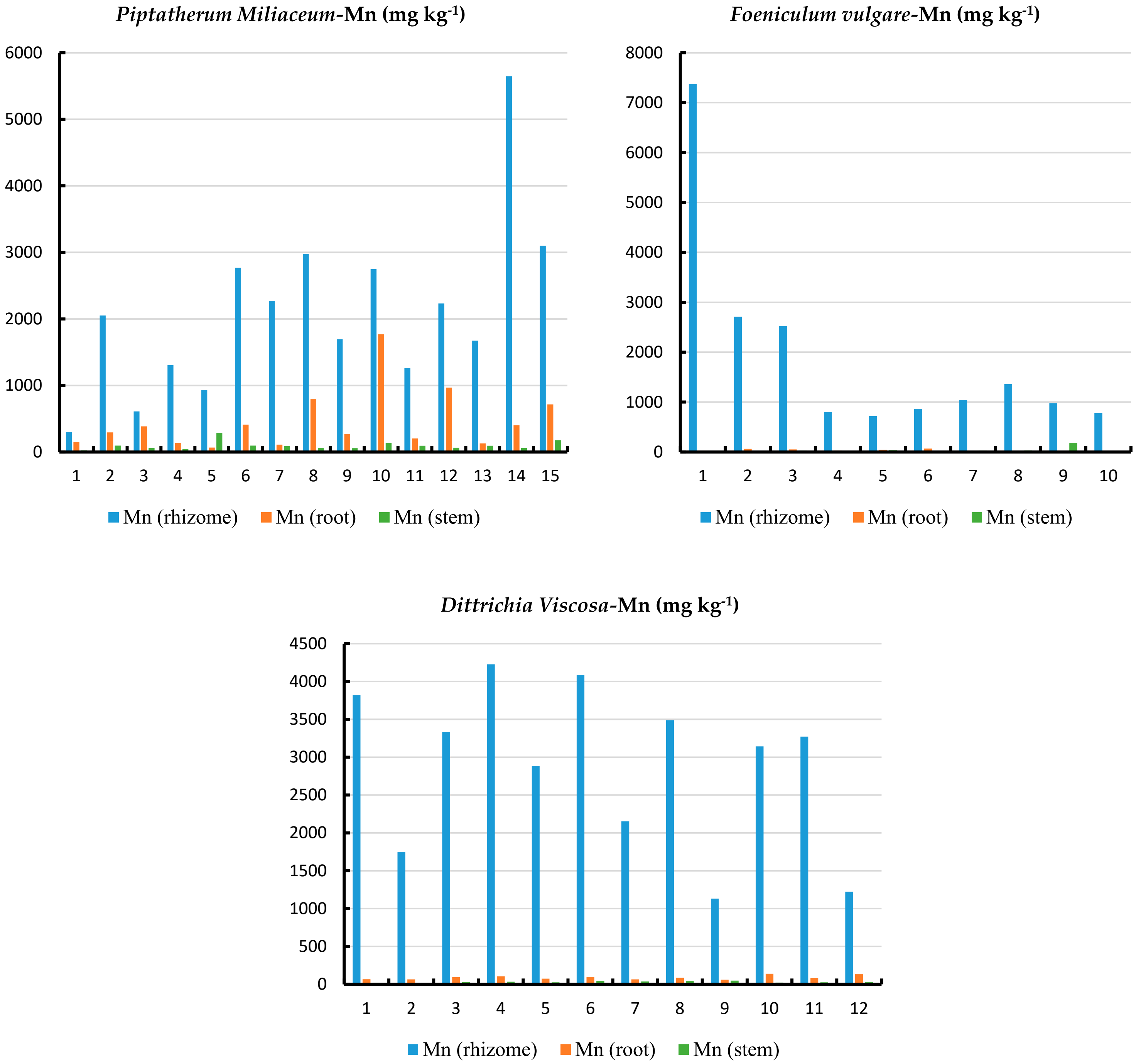

Figure S6. Mn concentration on sample points.

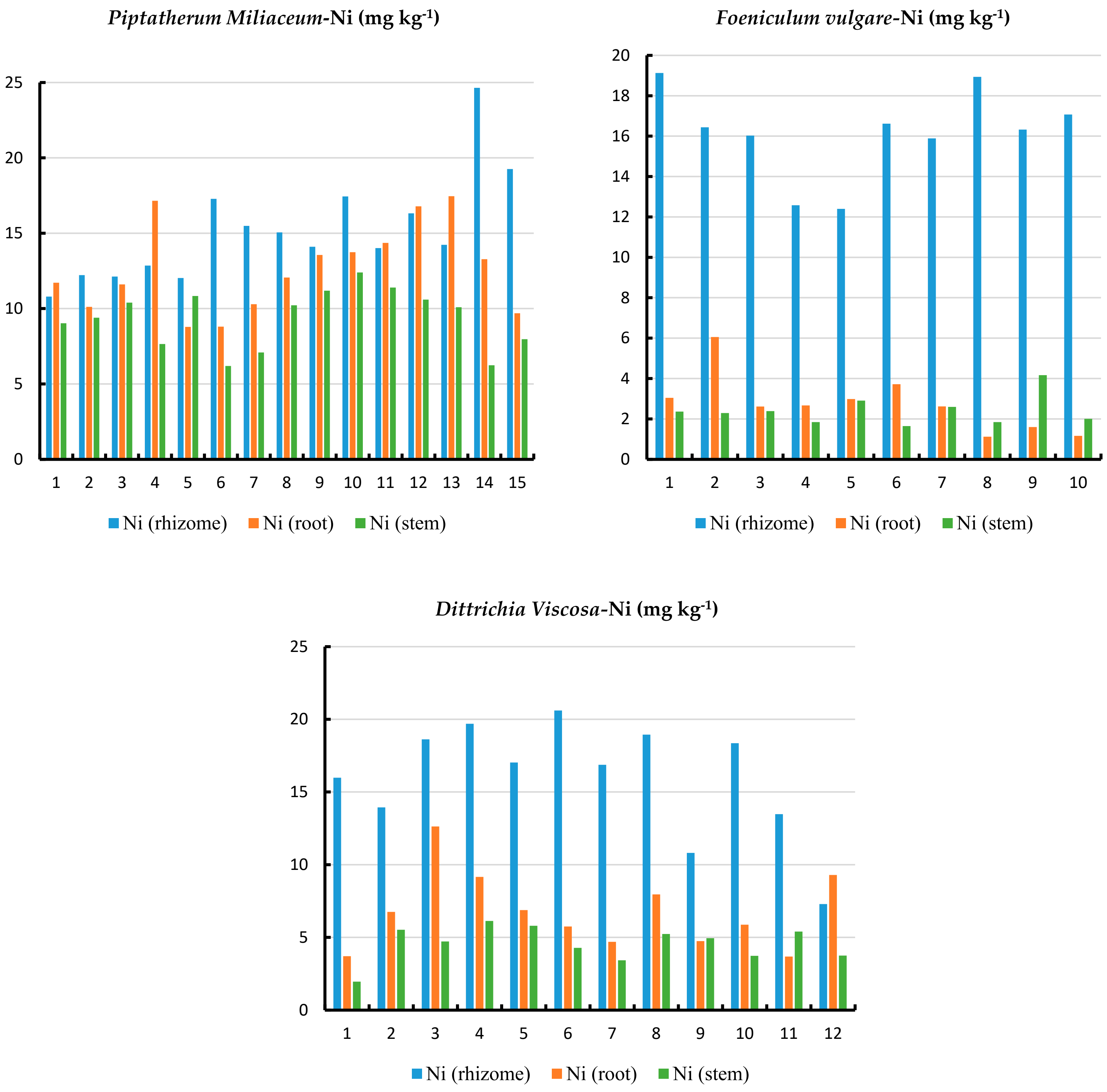

Figure S7. Ni concentration on sample points.

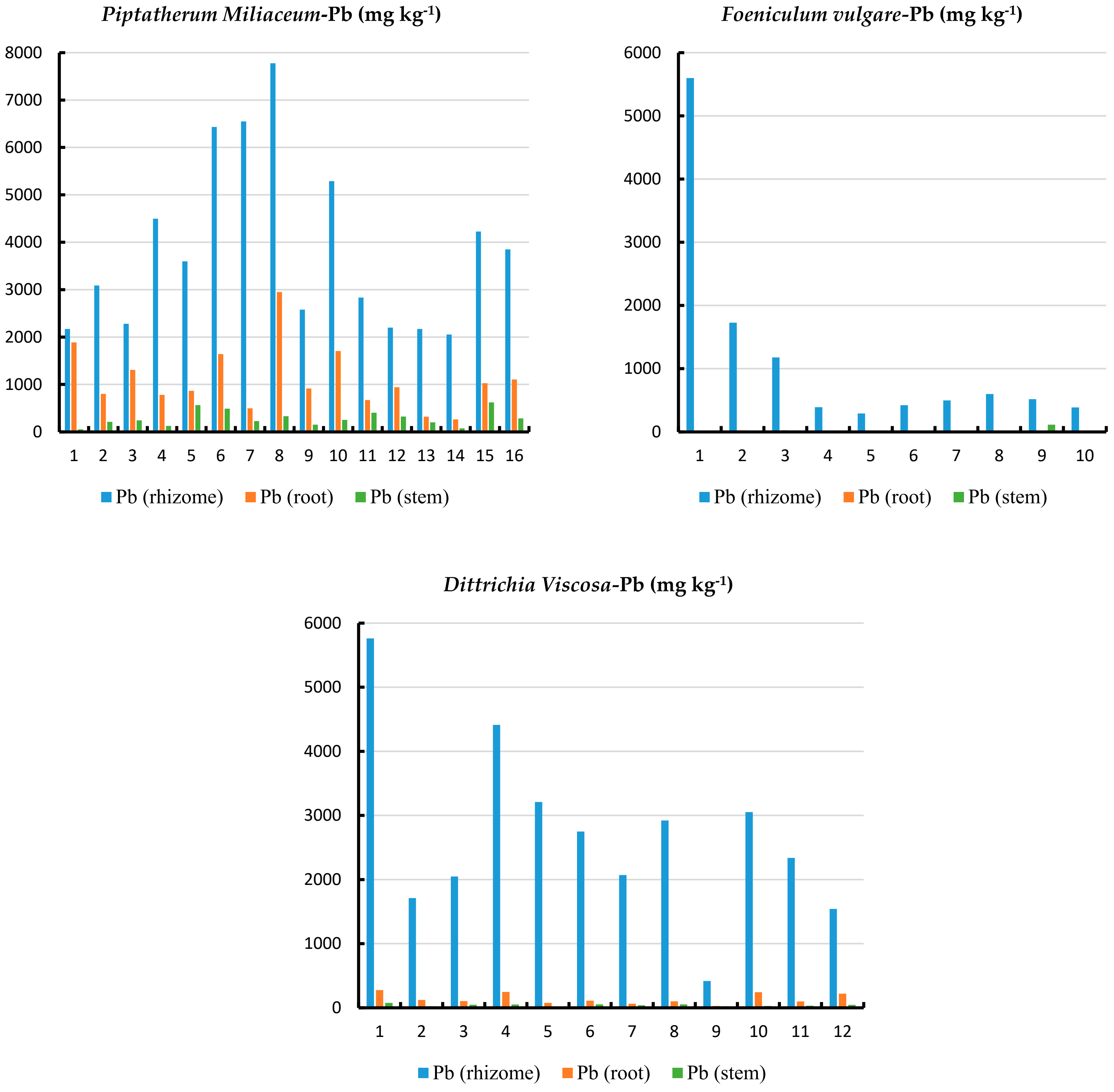

Figure S8. Pb concentration on sample points.

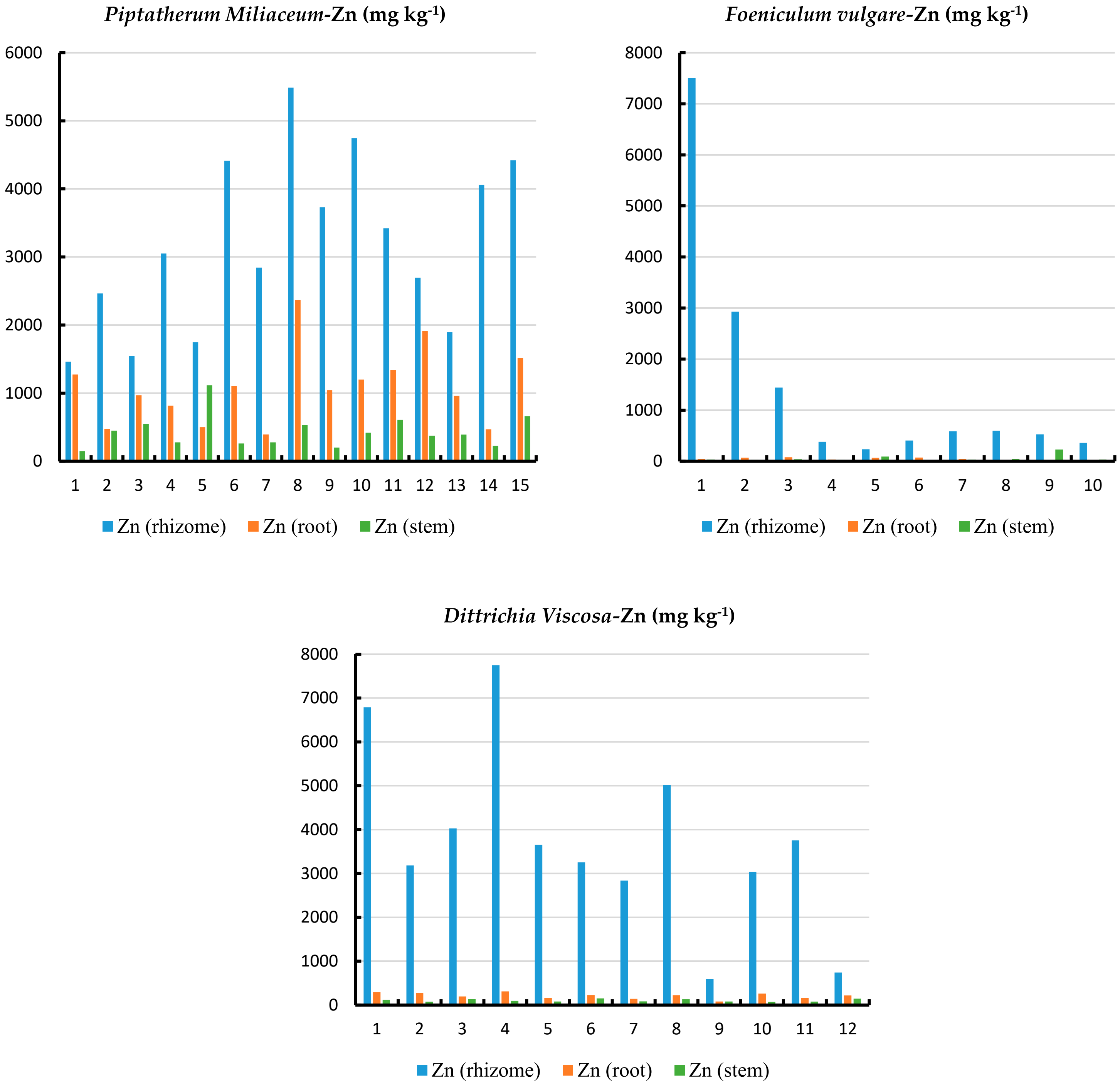

Figure S9. Zn concentration on sample points.

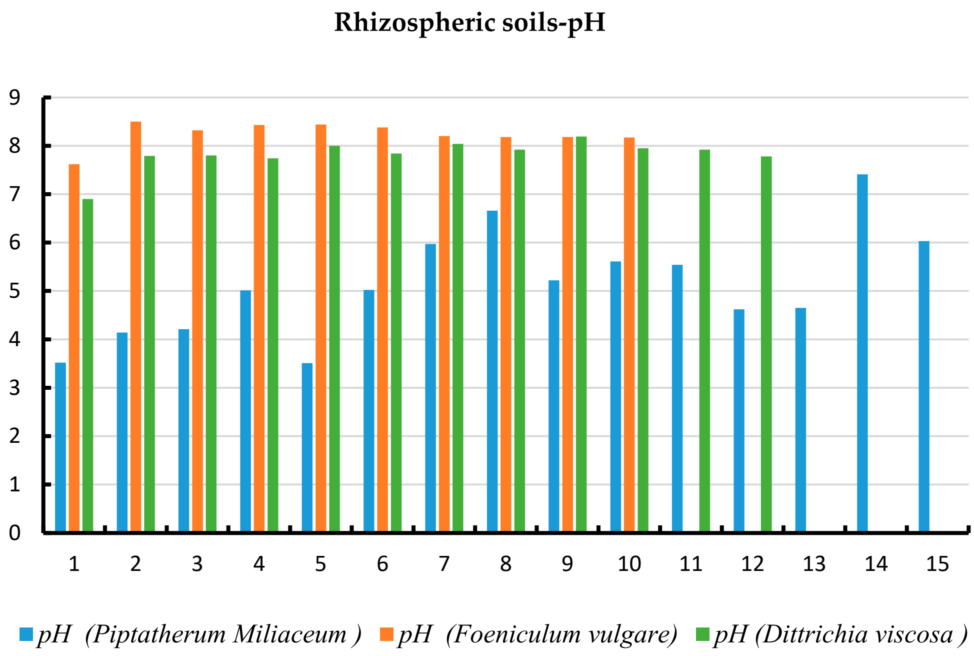

Figure S10. pH on sample points.

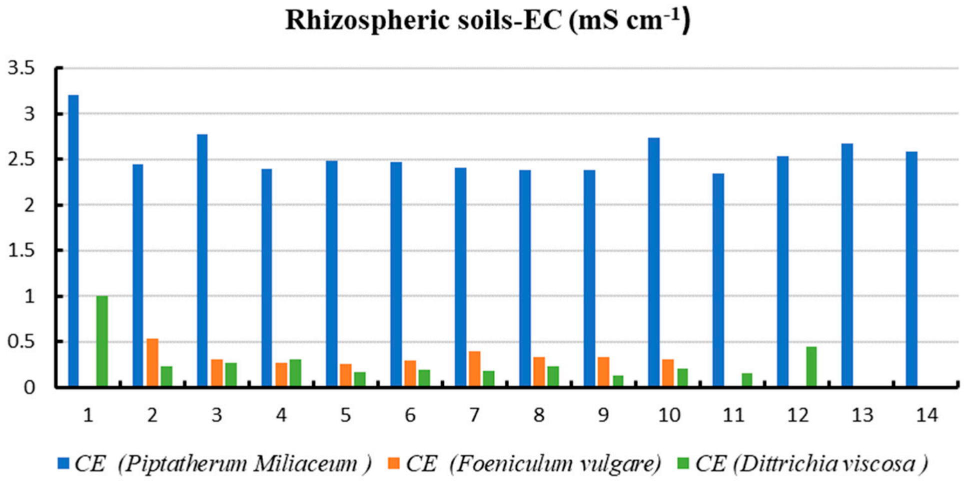

Figure S11. EC on sample points.

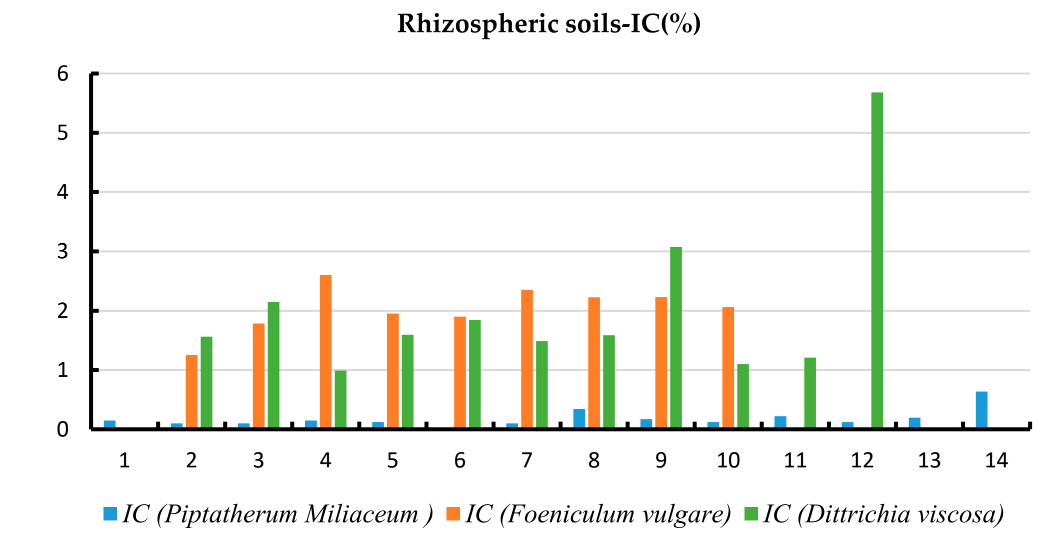

Figure S12. IC percentage on sample points.

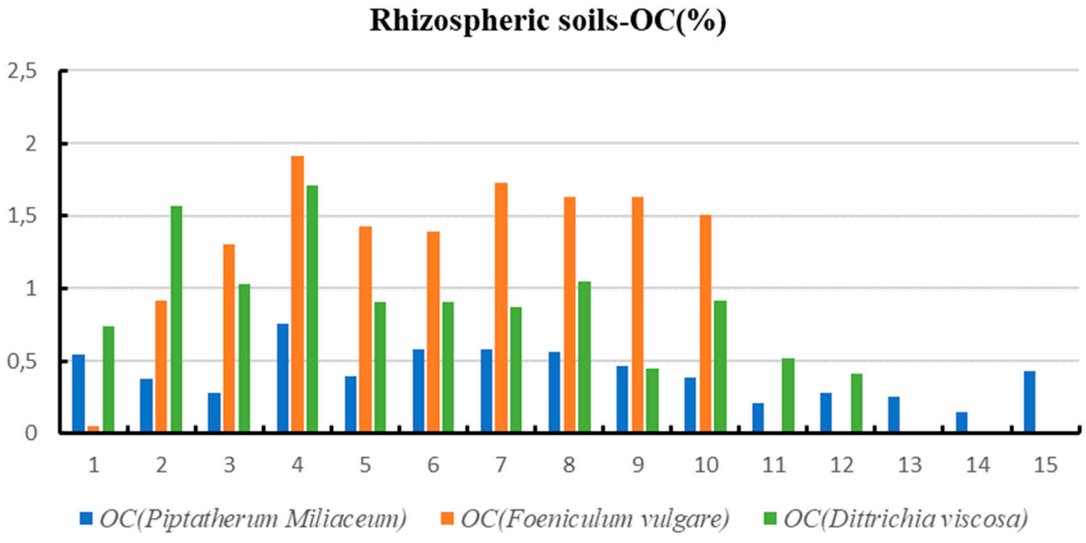

Figure S13. OC percentage on sample points.

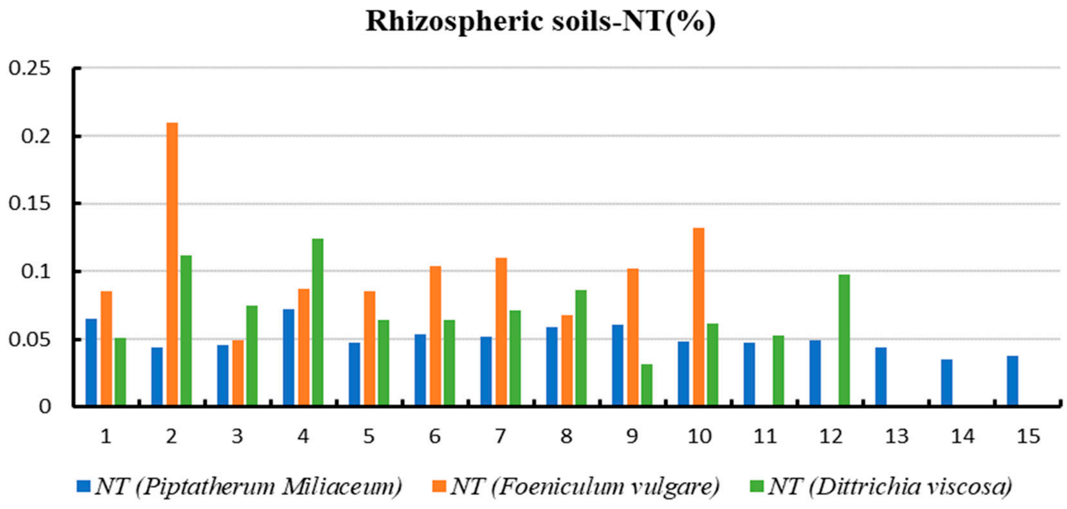

Figure S14. NT percentage on sample points.

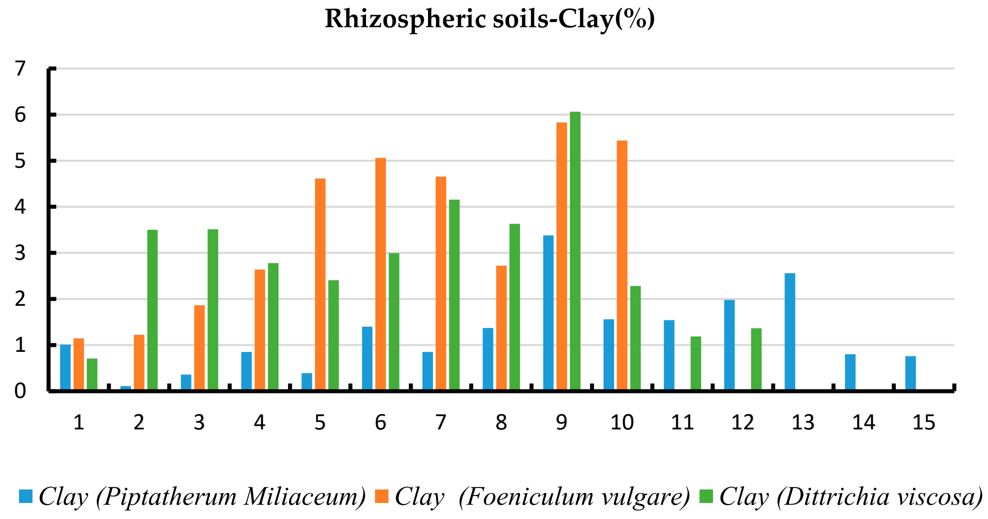

Figure S15. Clay percentage on sample points.

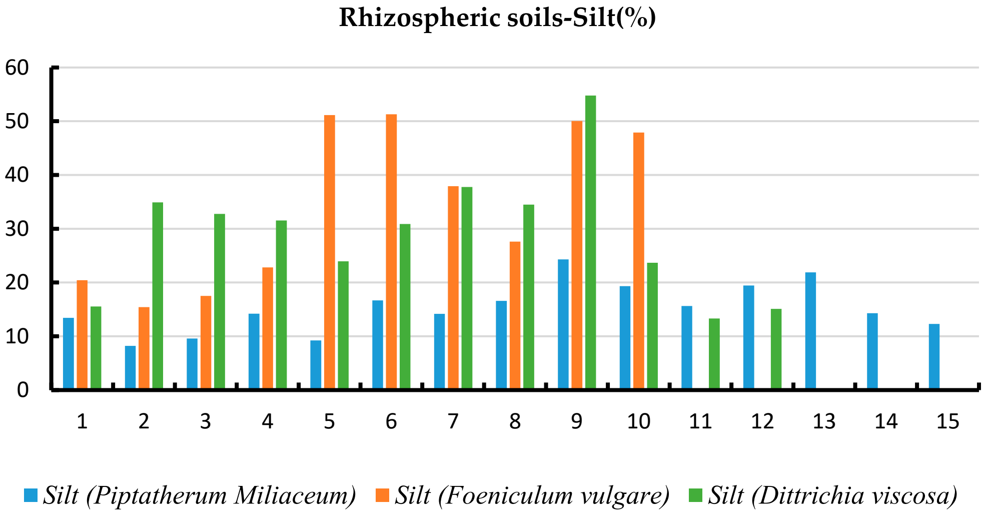

Figure S16. Silt percentage on sample points.

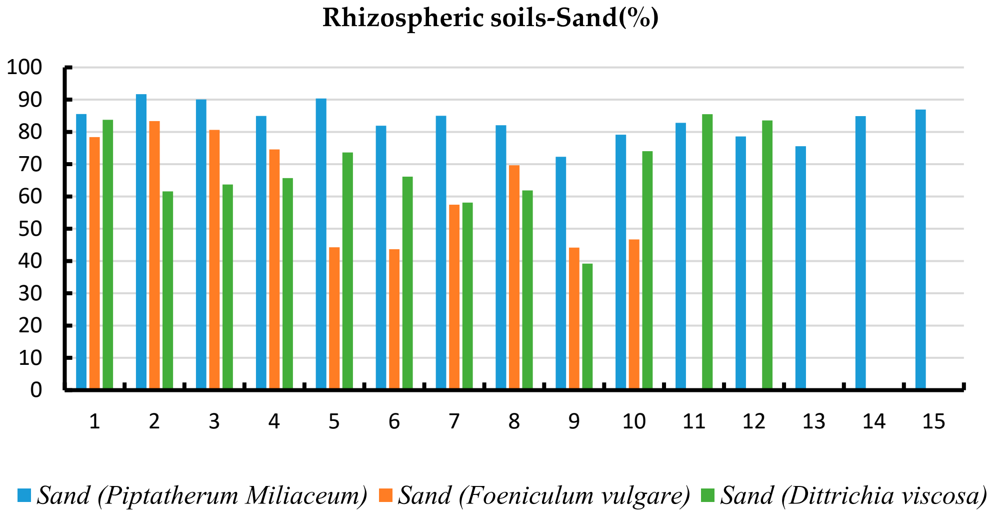

Figure S17. Sand percentage on sample points.

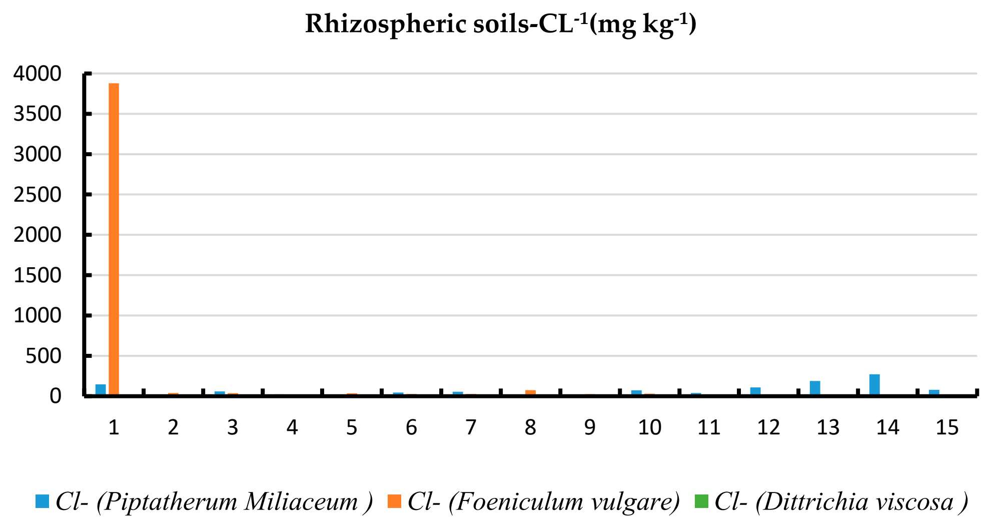

Figure S18. CL^- concentration on sample points.

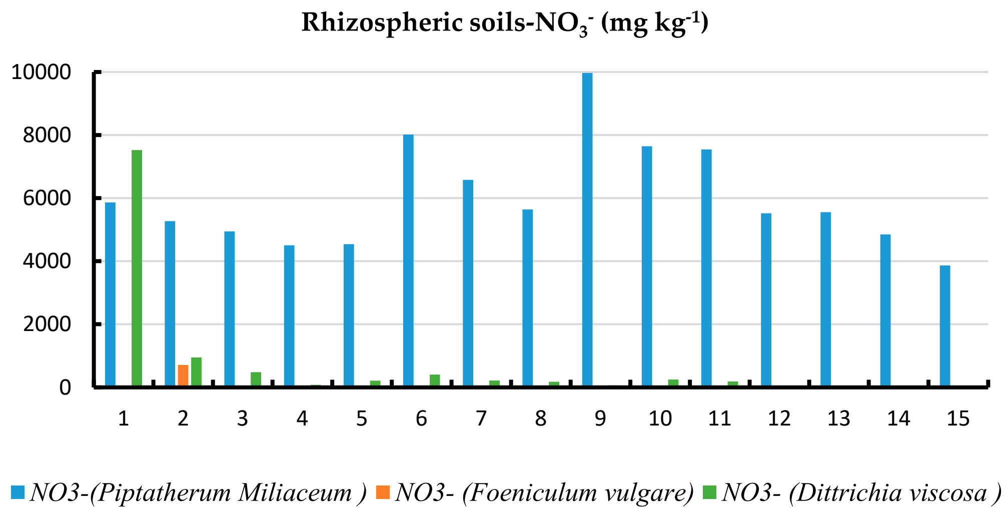

Figure S19. NO_3^- concentration on sample points.

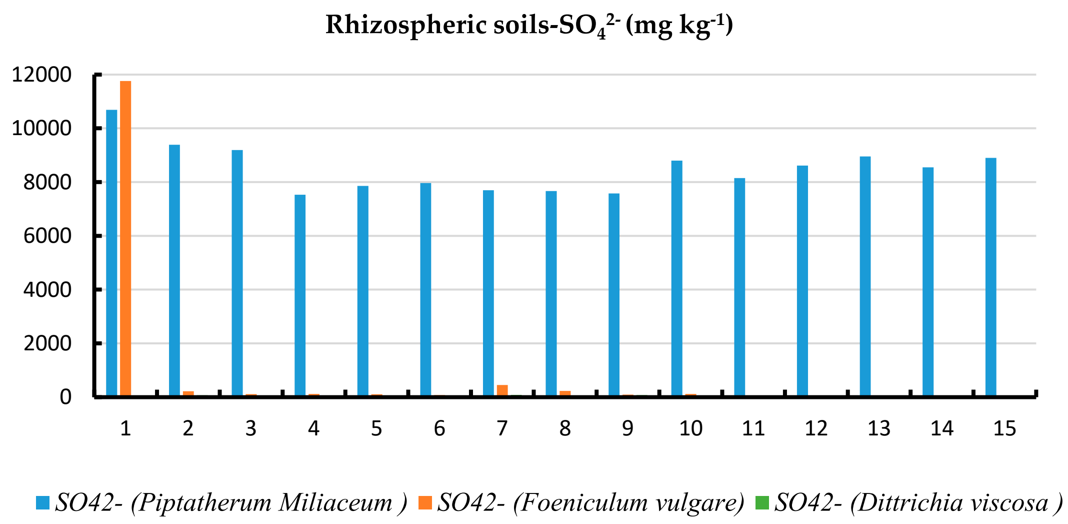

Figure S20. SO_4^{-2}concentration on sample points.

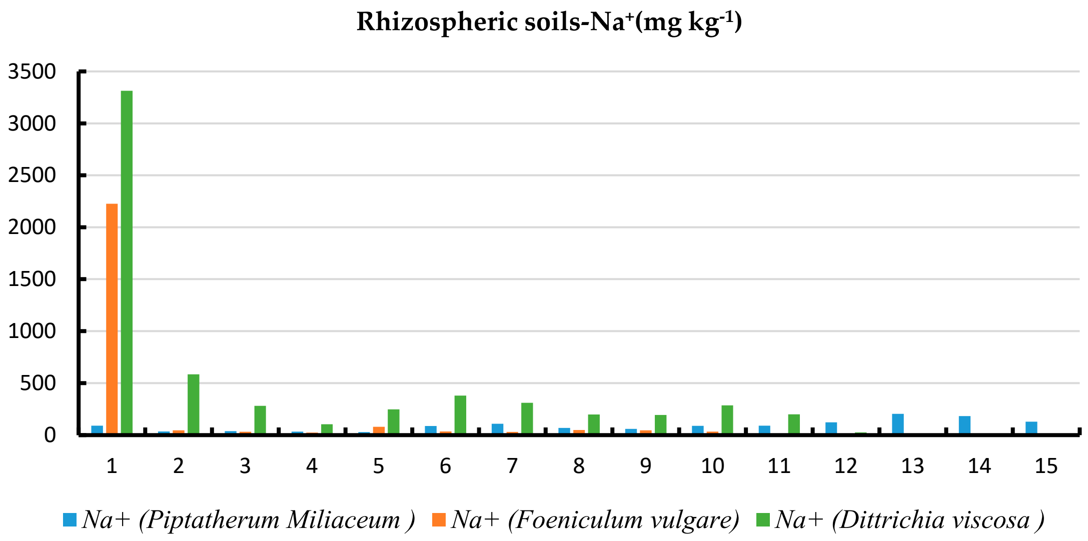

Figure S21. Na^{+} concentration on sample points.

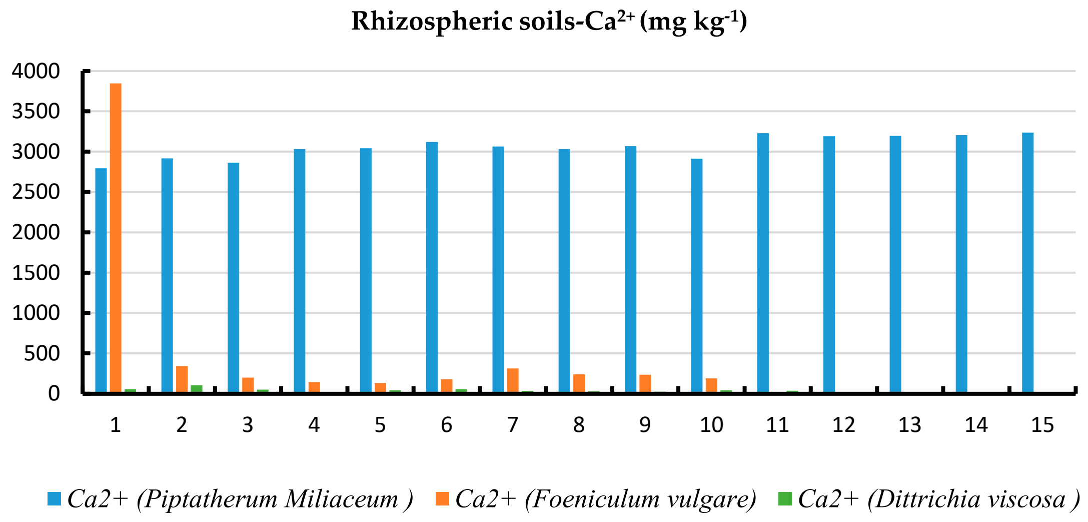

Figure S22. Ca^{2+} concentration on sample points.

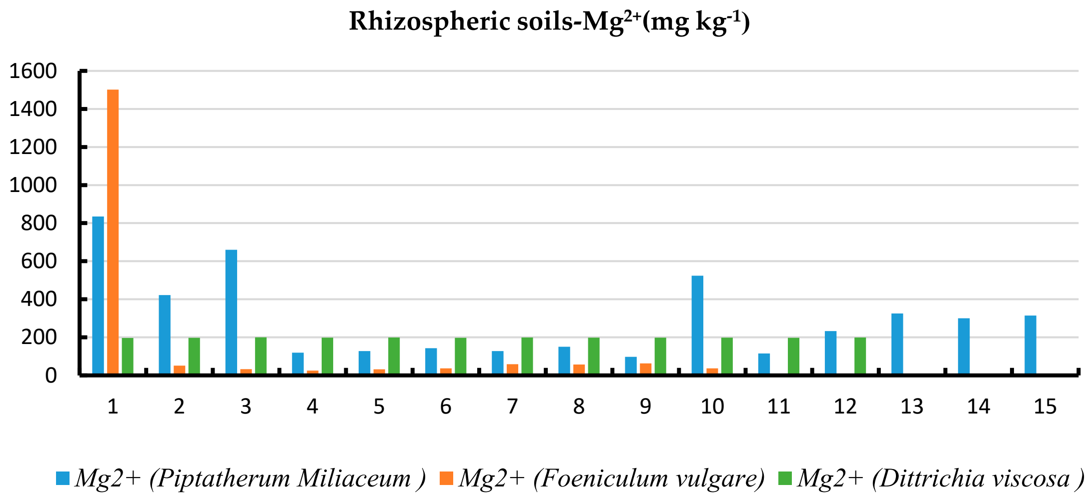

Figure S23. Mg^{2+} concentration on sample points.

Table S1. Correlation between total metal(loid) concentration in rhizospheric soil and metal(loid) concentration in the root (*Piptatherum Miliaceum*).

Metal (loid)s	As	Cd	Cr	Cu	Fe	Mn	Ni	Pb	Zn
r	0.23	0.61	0.10	0.42	0.28	0.61	0.09	0.31	0.35
p	0.41	0.02	0.72	0.12	0.31	0.02	0.76	0.26	0.20

Table S2. Correlation between bioavailable metal(loid) concentration in rhizospheric soil and metal(loid) concentration in the root (*Piptatherum Miliaceum*).

Metal (loid)s	As	Cd	Cr	Cu	Fe	Mn	Ni	Pb	Zn
r	-0.24	0.51	-0.28	0.05	-0.36	-0.06	0.21	0.15	0.22
p	0.40	0.05	0.31	0.85	0.18	0.82	0.44	0.60	0.44

Table S3. Correlation between total metal(loid) concentration in rhizospheric soil and metal(loid) concentration in the stem (*Piptatherum Miliaceum*).

Metal (loid)s	As	Cd	Cr	Cu	Fe	Mn	Ni	Pb	Zn
r	-0.49	0.38	-0.25	0.08	0.02	0.25	-0.26	0.51	0.03
p	0.06	0.16	0.38	0.79	0.94	0.36	0.34	0.05	0.92

Table S4. Correlation between bioavailable metal(loid) concentration in rhizospheric soil and metal(loid) concentration in the stem (*Piptatherum Miliaceum*).

Metal (loid)s	As	Cd	Cr	Cu	Fe	Mn	Ni	Pb	Zn
r	-0.13	0.24	0.42	-0.18	-0.29	0.30	0.35	0.30	-0.30
p	0.65	0.40	0.12	0.52	0.30	0.28	0.20	0.28	0.27

Table S5. Correlation between root metal(loid) concentration and metal(loid) concentration in the stem (*Piptatherum Miliaceum*).

Metal (loid)s	As	Cd	Cr	Cu	Fe	Mn	Ni	Pb	Zn
r	-0.09	0.50	0.16	0.13	0.08	0.30	0.31	0.29	0.20
p	0.76	0.05	0.58	0.66	0.78	0.28	0.27	0.29	0.47

Table S6. Correlation between total metal(loid) concentration in rhizospheric soil and metal(loid) concentration in the root (*Foeniculum vulgare*).

Metal (loid)s	As	Cd	Cr	Cu	Fe	Mn	Ni	Pb	Zn
r	0.50	0.18	0.03	-0.13	0.19	0.29	-0.12	0.16	0.38
p	0.14	0.63	0.93	0.73	0.60	0.43	0.75	0.65	0.28

Table S7. Correlation between bioavailable metal(loid) concentration in rhizospheric soil and metal(loid) concentration in the root (*Foeniculum vulgare*).

Metal (loid)s	As	Cd	Cr	Cu	Fe	Mn	Ni	Pb	Zn
r	-0.10	0.07	0.02	-0.06	0.06	-0.15	-0.46	-0.12	0.43
p	0.78	0.86	0.96	0.88	0.88	0.68	0.19	0.75	0.21

Table S8. Correlation between total metal(loid) concentration in rhizospheric soil and metal(loid) concentration in the stem (*Foeniculum vulgare*).

Metal (loid)s	As	Cd	Cr	Cu	Fe	Mn	Ni	Pb	Zn
r	0.12	-0.14	-0.16	-0.01	0.07	-0.18	-0.47	-0.16	-0.01
p	0.75	0.70	0.65	0.99	0.86	0.63	0.17	0.65	0.99

Table S9. Correlation between bioavailable metal(loid) concentration in rhizospheric soil and metal(loid) concentration in the stem (*Foeniculum vulgare*).

Metal (loid)s	As	Cd	Cr	Cu	Fe	Mn	Ni	Pb	Zn

r	-0.44	-0.06	-0.19	0.19	0.13	0.32	-0.14	0.10	0.14
p	0.20	0.88	0.60	0.60	0.73	0.37	0.70	0.78	0.70

Table S10. Correlation between root metal(loid) concentration and metal(loid) concentration in the stem (*Foeniculum vulgare*).

Metal (loid)s	As	Cd	Cr	Cu	Fe	Mn	Ni	Pb	Zn
r	-0.37	0.50	0.07	-0.32	-0.71	-0.41	-0.08	0.13	-0.43
p	0.29	0.05	0.86	0.37	0.02	0.24	0.83	0.73	0.21

Table S11. Correlation between total metal(loid) concentration in rhizospheric soil and metal(loid) concentration in the root (*Dittrichia Viscosa*).

Metal (loid)s	As	Cd	Cr	Cu	Fe	Mn	Ni	Pb	Zn
r	0.57	0.43	0.14	0.04	0.50	0.41	0.30	0.47	0.55
p	0.05	0.17	0.67	0.91	0.10	0.19	0.34	0.12	0.06

Table S12. Correlation between bioavailable metal(loid) concentration in rhizospheric soil and metal(loid) concentration in the root (*Dittrichia Viscosa*).

Metal (loid)s	As	Cd	Cr	Cu	Fe	Mn	Ni	Pb	Zn
r	0.20	0.39	0.08	-0.18	0.21	0.32	0.50	0.64	0.50
p	0.54	0.21	0.81	0.57	0.51	0.32	0.10	0.02	0.10

Table S13. Correlation between total metal(loid) concentration in rhizospheric soil and metal(loid) concentration in the stem (*Dittrichia Viscosa*).

Metal (loid)s	As	Cd	Cr	Cu	Fe	Mn	Ni	Pb	Zn
r	0.41	0.13	-0.18	-0.06	0.20	0.03	0.15	0.40	0.24
p	0.19	0.68	0.59	0.85	0.54	0.93	0.63	0.20	0.46

Table S14. Correlation between bioavailable metal(loid) concentration in rhizospheric soil and metal(loid) concentration in the stem (*Dittrichia Viscosa*).

Metal (loid)s	As	Cd	Cr	Cu	Fe	Mn	Ni	Pb	Zn
r	-0.32	0.11	0.25	0.07	-0.02	0.66	0.55	0.29	-0.03
p	0.30	0.73	0.44	0.83	0.95	0.02	0.07	0.35	0.93

Table S15. Correlation between root metal(loid) concentration and metal(loid) concentration in the stem (*Dittrichia Viscosa*).

Metal (loid)s	As	Cd	Cr	Cu	Fe	Mn	Ni	Pb	Zn
r	0.44	0.85	0.29	0.53	0.01	-0.06	0.33	0.51	0.08
p	0.15	0.00	0.37	0.08	0.98	0.86	0.30	0.09	0.80

Table S16. Mean values of individual potential ecological risk E_{ri} and total ecological risk RI (rhizospheric total concentrations) for the dry riverbeds.

E_{ri}	As	Cd	Cr	Cu	Mn	Ni	Pb	Zn	RI
EB	422	738	1.39	31.2	2.73	3.50	2069	77.3	3346
	(96)	(373)	(0.16)	(7.8)	(1.69)	(0.81)	(1010)	(30.4)	(1283)
LC	94	469	0.68	9.76	2.49	3.72	624	36.1	1240
	(207)	(733)	(0.10)	(7.79)	(2.66)	(0.51)	(872)	(54.6)	(1872)

PN	68<br>(74)	1364<br>(768)	0.76<br>(0.16)	11.5<br>(6.59)	3.73<br>(1.39)	3.68<br>(0.91)	1444<br>(747)	89.9<br>(50.4)	2986<br>(1543)

Table S17. Mean values of bioaccumulation factor BF_b (rhizospheric bioavailable concentrations) for the dry riverbeds.

BF_b	As	Cd	Cr	Cu	Fe	Mn	Ni	Pb	Zn
EB	1510<br>(1963)	4.26<br>(3.90)	6858<br>(3206)	10.5<br>(5.9)	294570<br>(747244)	198<br>(546)	724<br>(1877)	1.90<br>(1.51)	3.91<br>(3.34)
LC	16.6<br>(16.6)	0.98<br>(1.05)	1608<br>(1412)	2.43<br>(1.34)	62.6<br>(55.6)	1.08<br>(1.12)	10.5<br>(9.2)	0.13<br>(0.08)	1.25<br>(1.22)
PN	144<br>(177)	0.71<br>(0.23)	2280<br>(1293)	6.01<br>(5.54)	355<br>(162)	2.07<br>(2.02)	53.3<br>(36.4)	1.22<br>(1.06)	3.45<br>(1.27)

Table S18. Mean values of mobility ratio MR_b (rhizospheric bioavailable concentrations) for the dry riverbeds.

MR_b	As	Cd	Cr	Cu	Fe	Mn	Ni	Pb	Zn
EB	302<br>(560)	2.20<br>(2.41)	6858<br>(3206)	5.20<br>(3.31)	48138<br>(100679)	28.4<br>(77.4)	413<br>(900)	0.42<br>(0.41)	1.75<br>(1.67)
LC	34.6<br>(76.9)	0.76<br>(1.22)	1341<br>(1003)	2.63<br>(1.49)	69.8<br>(110.7)	0.89<br>(0.89)	8.14<br>(4.84)	0.19<br>(0.30)	1.62<br>(1.99)
PN	45.9<br>(54.1)	0.24<br>(0.13)	1369<br>(435)	3.63<br>(2.16)	108<br>(55)	2.16<br>(2.06)	34.3<br>(13.6)	0.70<br>(0.77)	1.19<br>(0.35)
